# Synthesis of novel pyrroles and fused pyrroles as antifungal and antibacterial agents

**DOI:** 10.1080/14756366.2021.1984904

**Published:** 2021-10-04

**Authors:** Rania Helmy Abd El-Hameed, Amira Ibrahim Sayed, Shima Mahmoud Ali, Mohamed A. Mosa, Zainab M. Khoder, Samar Said Fatahala

**Affiliations:** a Pharmaceutical Organic Chemistry Department, Faculty of Pharmacy, Helwan University, Helwan, Cairo, Egypt; b Microbiology and Immunology Department, Faculty of Pharmacy, Helwan University, Helwan, Cairo, Egypt; c Plant Pathology Research Institute, Agricultural Research Center, Giza, Egypt; d Department of Chemistry, The state University of New York at Buffalo, New York, NY, USA

**Keywords:** Pyrroles, fused pyrroles, synthesis, anti-bacterial, anti-fungal

## Abstract

Pyrroles and its fused forms possess antimicrobial activities, they can easily interact with biomolecules of living systems. A series of substituted pyrroles, and its fused pyrimidines and triazines forms have been synthesised, all newly synthesised compound structures were confirmed by spectroscopic analysis. Generally, the compounds inhibited growth of some important human pathogens, the best effect was given by: **2a, 3c, 4d** on Gram-positive bacteria and was higher on yeast (*C. albican*s), by **5c** on Gram-negative bacteria and by **5a then 3c** on filamentous fungi *(A. fumigatus* and *F. oxysporum*). Such results present good antibacterial and antifungal potential candidates to help overcome the global problem of antibiotic resistance and opportunistic infections outbreak. Compound **3c** gave the best anti-phytopathogenic effect at a 50-fold lower concentration than Kocide 2000, introducing a safe commercial candidate for agricultural use. The effect of the compounds on DNA was monitored to detect the mode of action.

## 1. Introduction

Pathogenic bacteria are a major public health burden because they cause morbidity, mortality, and moreover increased healthcare costs. Moreover, the rapid evolution of resistance to antimicrobial agents puts an extra load^1^. Some of the most serious and resistant organisms are *Staphylococcus aureus* (*S. aureus*)^1^ and *Escherichia coli (E. coli)* O157:H7 that belongs to the enterohemorrhagic *E. coli* (EHEC) class^2^, and *Pseudomonas aeruginosa (P. aeruginosa*)^3^ that represents the most common cause of nosocomial infections (OIs)^4^.

**Figure 1.**
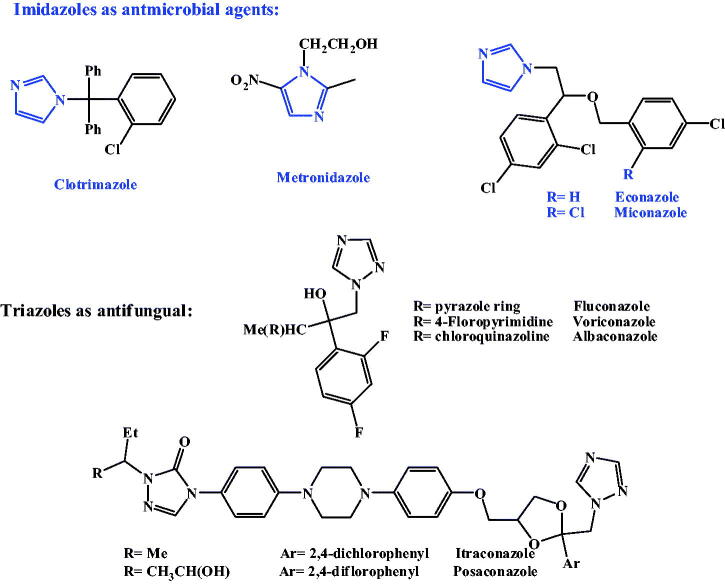
Azoles and triazoles as antimicrobial agents.

Another major cause of OIs and the consequent fatality is the fungal pathogens. The most common opportunistic fungi include Candida species, Aspergillus species, and Fusarium species^5^. Candida species, was a major cause of morbidity and mortality worldwide over the past few decades^6^. *Aspergillus fumigatus (A. fumigatus*) is a saprophyte that has become the most prevalent airborne fungal pathogen^7^. *A. fumigatus* causes severe and usually fatal invasive aspergillosis in immuno-compromised hosts in developed countries^7^. The members of *Fusarium oxysporum (F. oxysporum*) complex, (FOSC) are soil borne pathogens that cause diseases to a broad range of important crops with a number of them having the ability to cause human diseases that can be fatal^8^. Because fungi are eukaryotes, they have similarities with mammalian cells making it difficult to design a drug with selective toxicity^9^. The first antifungal drug discovered was **amphotericin B**^10^ followed by **flucytosine**^11^, later on two imidazole derivatives, **clotrimazole** and **miconazole**, were extensively used in treatment of fungal infections^12–15^. Triazole derivative, **fluconazole**, is an antifungal agent with low toxicity and high antifungal activity^16^. Resistance of some fungi against imidazole is now becoming a serious clinical problem^17^, several azole-related adverse drug effects (ADEs) are now considered such as hepatotoxicity and cardiac effects. In order to enrich the antifungal activity and/or diminish antifungal ADEs, new derivatives were synthesised containing other heterocycles instead of imidazole and 1,2,4-triazole, such as indole, 1,2,3-triazoles^14^^,^^18–20^. **Voriconazole** is a clinically important triazoles antifungal containing fluoropyrimidine^13^^,^^20^. Using quinazoline ring instead of a triazole ring results in **albaconazole** with higher *in vitro* activity than **fluconazole**^21^. **Itraconazole** with piperazinyl phenyl side chain showed remarkable *in vitro* antifungal activity against several pathogenic fungi^16^, as shown in Figure 1.

**Scheme 1.**
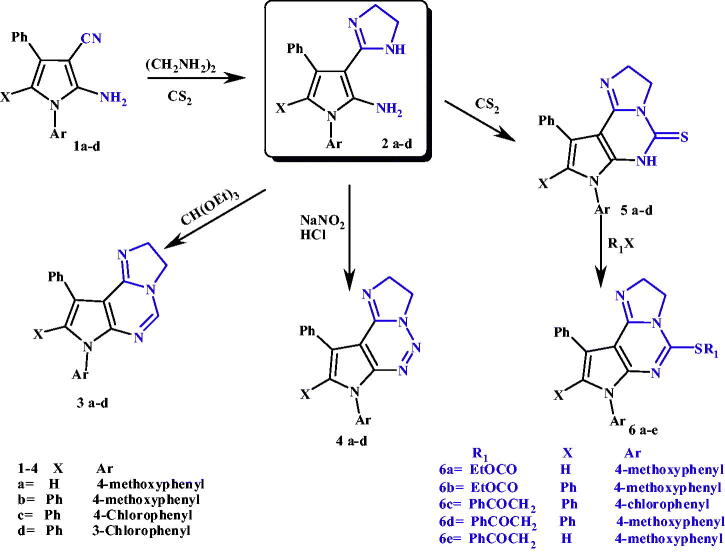
Synthesis of targeted compounds 2–6.

**Figure 2.**
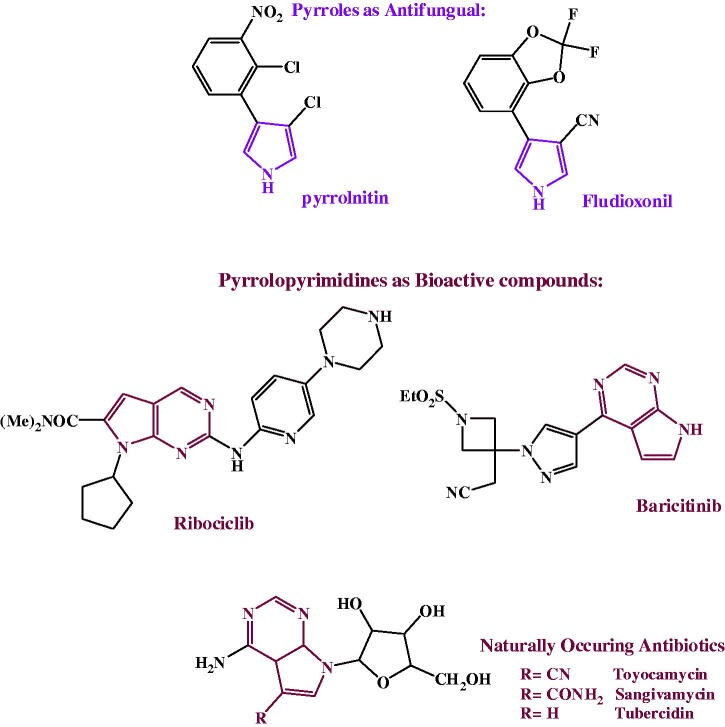
pyrroles and pyrrolopyrimidines as bioactive compounds.

Pyrroles and its fused derivatives, are an important class of naturally^22^^,^^23^ and synthetically^24–26^ occurring compounds with a wide-range of biological activities; antibacterial^27–29^, antifungal^30–33^, antiviral^34–39^, anticancer^40^^,^^41^ and anti-inflammatory^42^^,^^43^. Pyrrolnitin and fludioxonil, are two naturally secreted pyrroles, reported to bear a broad spectrum antifungal activities^44–46^. 7-deazapurine, naturally secreted pyrrolopyrimidines antibiotics, toyocamycin, tubercidin and sangivamycin, commonly have antibacterial, antifungal, anticancer, antiviral and anti-inflammatory activities^22^^,^^47^. Due to structural resemblance to purine, 7-deazapurines interfered with various cellular processes; toyocamycin united with tRNA, pyrrolopyrimidine inhibits tRNA aminoacylation. Sangivamycin has lately been revealed to inhibit protein kinases^22^, as revealed in Figure 2.

Health organisations, such as the WHO, CDC and FDA are considering the growing antimicrobial resistance a potential threat for human health^48^. Fungi also are showing a growing resistance with increased number of fatal systemic mycoses^9^. That’s why the discovery of new effective antifungal and antibacterial drugs has become a high priority, and this is the aim of our study through preparation and testing of novel 3-imidazolinyl pyrroles, its fused form (namely; pyrimidnes and triazines).

## 2. Materials and methods

### 2.1. Synthesis of lead compounds

All commercial chemicals used as starting materials and reagents in this study were purchased from Merck (Darmstadt, Germany) and were of reagent grade. All melting points were uncorrected and measured using Electro-thermal IA 9100 apparatus (Shimadzu, Japan); IR spectra were recorded as potassium bromide pellets on a Perkin-Elmer 1650 spectrophotometer (USA), Faculty of Science, Cairo University, Cairo, Egypt. ^1^H-NMR spectra were determined on a Varian Mercury (300 MHz) spectrometer (Varian UK) and chemical shifts were expressed as ppm against TMS as internal reference (The Main Chemical Warfare Laboratories, Almaza, Cairo, Egypt). Mass spectra were recorded on 70 eV (EI Ms-QP 1000 EX, Shimadzu, Japan), Faculty of Science, Cairo University, Cairo, Egypt. Microanalyses were operated using Vario, Elmentar apparatus (Shimadzu, Japan), Organic Microanalysis Unit, Faculty of Science, Cairo University, Cairo, Egypt. Column Chromatography was performed on (Merck) Silica gel 60 (particle size 0.06-0.20 mm). All the listed compounds are new except compounds **1a-d** was previously reported^38^^,^^49–51^.

#### General procedure for the synthesis of compounds 2a-d:

To a mixture of **1a-d** (5 mmol) and ethylenediamine (7.5 mL) carbon disulphide (0.7 mL) was added dropwise, and the reaction mixture was heated under reflux for 4 h. After cooling the reaction mixture was poured into cold water and the precipitate obtained was filtered off, washed with water, dried and crystallized from ethanol to yield compounds **2a-d**

***3–(4,5-Dihydro-1H-imidazol-2-yl)-1–(4-methoxyphenyl)-4-phenyl-pyrrol-2-amine (2a):*** Yield: 87%; m.p.: 187–189 °C; IR (KBr) υ (cm^−1^): 3347 (NH)), 3137 (NH_2_); MS (EI) *m*/*z*: 332 (M^+^, 34.24%); ^1^H-NMR (DMSO-d_6_, 300 MHz) δ (ppm): 3.02 (*t*, 2H, *J* = 8.1 Hz, CH_2_ imidazoline), 3.39 (*t*, 2H, *J* = 8.1 Hz, CH_2_ imidazoline), 3.45 (*s*, 3H, OCH_3_), 4.34 (*s*, 2H, NH_2_, D_2_O exchangeable), 6.72–8.47 (*m*, 10H, Ar-H), 9.64 (*s*, 1H, NH, D_2_O exchangeable); Anal. Calcd. for C_20_H_20_N_4_O (332.4): C, 72.29; H, 6.02; N, 16.87%. Found: C, 72.49; H, 6.31; N, 16.54%.

***3–(4,5-Dihydro-1H-imidazol-2-yl)-1–(4-methoxyphenyl)-4,5-diphenyl-pyrrol-2-amine (2 b):*** Yield: 81%; m.p.: 251–253 °C; IR (KBr) υ (cm^−1^): 3290 (NH)), 3122 (NH_2_); MS (EI) *m*/*z*: 408 (M^+^, 67.19%); ^1^H-NMR (DMSO-d_6_, 300 MHz) δ (ppm): 3.37 (*t*, 2H, *J* = 8.4 Hz, CH_2_ imidazoline), 4.22 (*s*, 3H, OCH_3_), 4.57 (*t*, 2H, *J* = 8.4 Hz, CH_2_ imidazoline), 5.72 (*s*, 2H, NH_2_, D_2_O exchangeable), 7.01–8.37 (*m*, 14H, Ar-H), 8.84 (*s*, 1H, NH, D_2_O exchangeable); Anal. Calcd. for C_26_H_24_N_4_O (408.4): C, 76.47; H, 5.88; N, 13.73%. Found: C, 76.29; H, 6.03; N, 13.74%.

***1–(4-Chlorophenyl)-3–(4,5-dihydro-1H-imidazol-2-yl)-4,5-diphenyl-pyrrol-2-amine (2c):*** Yield: 77%; m.p.: 210–212 °C; IR (KBr) υ (cm^−1^): 3403 (NH)), 3282 (NH_2_); MS (EI) *m*/*z*: 412 (M^+^, 19.2%); ^1^H-NMR (DMSO-d_6_, 300 MHz) δ (ppm): 2.53 (*t*, 2H, *J* = 8.2 Hz, CH_2_ imidazoline), 3.44 (*t*, 2H, *J* = 8.2 Hz, CH_2_ imidazoline), 5.20 (*s*, 2H, NH_2_, D_2_O exchangeable), 6.53–7.92 (*m*, 14H, Ar-H), 9.05 (*s*, 1H, NH, D_2_O exchangeable); Anal. Calcd. for C_25_H_21_ClN_4_ (412.14): C, 72.82; H, 5.10; N, 13.59%. Found: C, 73.08; H, 5.07; N, 13.66%.

***1–(3-Chlorophenyl)-3–(4,5-dihydro-1H-imidazol-2-yl)-4,5-diphenyl-pyrrol-2-amine (2d):*** Yield: 71%; m.p.: 205–207 °C; IR (KBr) υ (cm^−1^): 3337 (NH)), 3271 (NH_2_); MS (EI) *m*/*z*: 412 (M^+^, 22.52%); ^1^H-NMR (DMSO-d_6_, 300 MHz) δ (ppm): 2.53 (*t*, 2H, *J* = 8.4 Hz, CH_2_ imidazoline), 3.51 (t, 2H, *J* = 8.4 Hz, CH_2_ imidazoline), 5.87 (*s*, 2H, NH_2_, D_2_O exchangeable), 6.97–8.14 (*m*, 14H, Ar-H), 8.94 (*s*, 1H, NH, D_2_O exchangeable); Anal. Calcd. for C_25_H_21_ClN_4_ (412.14): C, 72.82; H, 5.10; N, 13.59%. Found: C, 72.94; H, 5.18; N, 13.34%.

#### General procedure for the synthesis of compounds 3a-d:

A mixture of **2a-d** (5 mmol) and triethyl orthoformate (15 ml) was heated under reflux for 3 h. After cooling, the reaction mixture was poured into cold water. The solid precipitate was filtered off, washed with water, dried and recrystallized from dioxane to give **3a-d**

***7–(4-methoxyphenyl)-9-phenyl-3,7-dihydro-2H-imidazo[1,2-c]pyrrolo[3,2-e]pyrimidine (3a):*** Yield: 90%; m.p.: 138–140 °C; IR (KBr) υ (cm^−1^): 1596 (C = N); MS (EI) *m*/*z*: 342 (M^+^, 72.03%); ^1^H-NMR (DMSO-d_6_, 300 MHz) δ (ppm): 3.43 (*t*, 2H, *J* = 7.3 Hz, CH_2_ imidazoline), 3.97 (*s*, 3H, OCH_3_), 4.14 (*t*, 2H, *J* = 7.3 Hz, CH_2_ imidazoline), 6.91–7.34 (*m*, 11H, Ar-H); Anal. Calcd. for C_21_H_18_N_4_O (342.14): C, 73.68; H, 5.26; N, 16.37%. Found: C, 73.43; H, 5.11; N, 16.61%.

***7–(4-methoxyphenyl)-8,9-diphenyl-3,7-dihydro-2H-imidazo[1,2-c]pyrrolo[3,2-e]pyrimidine (3 b):*** Yield: 88%; m.p.: 193–195 °C; IR (KBr) υ (cm^−1^): 1580 (C = N); MS (EI) *m*/*z*: 418 (M^+^, 8.99%); ^1^H-NMR (DMSO-d_6_, 300 MHz) δ (ppm): 2.55 (*t*, 2H, *J* = 8.6 Hz, CH_2_ imidazoline), 2.63 (*t*, 2H, *J* = 8.6 Hz, CH_2_ imidazoline), 4.38 (*s*, 3H, OCH_3_), 6.73–8.16 (*m*, 15H, Ar-H); Anal. Calcd. for C_27_H_22_N_4_O (418.17): C, 77.51; H, 5.26; N, 13.40%. Found: C, 77.41; H, 5.36; N, 13.62%.

***7–(4-Chlorophenyl)-8,9-diphenyl-3,7-dihydro-2H-imidazo[1,2-c]pyrrolo[3,2-e]pyrimidine (3c):*** Yield: 82%; m.p.: 178–180 °C; IR (KBr) υ (cm^−1^): 1598 (C = N); MS (EI) *m*/*z*: 422 (M^+^, 10.37%); ^1^H-NMR (DMSO-d_6_, 300 MHz) δ (ppm): 3.36 (*t*, 2H, *J* = 7.4 Hz, CH_2_ imidazoline), 3.44 (*t*, 2H, *J* = 7.4 Hz, CH_2_ imidazoline), 6.61–7.82 (*m*, 15H, Ar-H); Anal. Calcd. for C_26_H_19_ClN_4_ (422.12): C, 73.93; H, 4.50; N, 13.27%. Found: C, 73.71; H, 4.44; N, 13.32%.

***7–(3-Chlorophenyl)-8,9-diphenyl-3,7-dihydro-2H-imidazo[1,2-c]pyrrolo[3,2-e]pyrimidine (3d):*** Yield: 75%; m.p.: 138–140 °C; IR (KBr) υ (cm^−1^): 1603 (C = N); MS (EI) *m*/*z*: 422 (M^+^, 54.17%); ^1^H-NMR (DMSO-d_6_, 300 MHz) δ (ppm): 3.15 (*t*, 2H, *J* = 7.3 Hz, CH_2_ imidazoline), 3.99 (*t*, 2H, *J* = 7.3 Hz, CH_2_ imidazoline), 7.69–8.08 (*m*, 15H, Ar-H); Anal. Calcd. for C_26_H_19_ClN_4_ (422.12): C, 73.93; H, 4.50; N, 13.27%. Found: C, 74.04; H, 4.38; N, 13.02%.

#### General procedure for the synthesis of compounds 4a-d:

To a cold solution of **2a-d** (5 mmol) in conc. hydrochloric acid (15 ml) and acetic acid (15 ml) a solution of sodium nitrite (2 g) in water (15 ml) was added portion-wise. After completion of the addition (30 min), the ice bath was removed and stirring was continued for 2 h. The solid product was filtered off and recrystallized from dioxane to afford **4a-d**

***7–(4-methoxyphenyl)-9-phenyl-3,7-dihydro-2H-imidazo[1,2-c]pyrrolo[3,2-e][1,2,3]triazine (4a):*** Yield: 85%; m.p.: 208–210 °C; IR (KBr) υ (cm^−1^): 1612 (C = N); MS (EI) *m*/*z*: 343 (M^+^, 18%); ^1^H-NMR (DMSO-d_6_, 300 MHz) δ (ppm): 3.18 (*t*, 2H, *J* = 8 Hz, CH_2_ imidazoline), 3.39 (*t*, 2H, *J* = 8 Hz, CH_2_ imidazoline), 4.55 (*s*, 3H, OCH_3_), 6.87–8.06 (*m*, 10H, Ar-H); Anal. Calcd. for C_20_H_17_N_5_O (343.14): C, 69.97; H, 4.96; N, 20.41%. Found: C, 70.04; H, 5.10; N, 20.23%.

***7–(4-methoxyphenyl)-8,9-diphenyl-3,7-dihydro-2H-imidazo[1,2-c]pyrrolo[3,2-e][1,2,3]triazine (4 b):*** Yield: 86%; m.p.: 204–206 °C; IR (KBr) υ (cm^−1^): 1592(C = N); MS (EI) *m*/*z*: 419 (M^+^, 33.5%); ^1^H-NMR (DMSO-d_6_, 300 MHz) δ (ppm): 3.28 (*t*, 2H, *J* = 7.6 Hz, CH_2_ imidazoline), 3.75 (*s*, 3H, OCH_3_), 4.23(t, 2H, *J* = 7.6 Hz, CH_2_ imidazoline), 6.71–8.32 (*m*, 14H, Ar-H); Anal. Calcd. for C_26_H_21_N_5_O (419.17): C, 74.46; H, 5.01; N, 16.71%. Found: C, 74.33; H, 5.12; N, 16.56%.

***7–(4-Chlorophenyl)-8,9-diphenyl-3,7-dihydro-2H-imidazo[1,2-c]pyrrolo[3,2-e][1,2,3]triazine (4c):*** Yield: 73%; m.p.: 159–161 °C; IR (KBr) υ (cm^−1^): 1595 (C = N); MS (EI) *m*/*z*: 423 (M^+^, 26.9%); ^1^H-NMR (DMSO-d_6_, 300 MHz) δ (ppm): 3.36 (*t*, 2H, *J* = 8.1 Hz, CH_2_ imidazoline), 3.43 (*t*, 2H, *J* = 8.1 Hz, CH_2_ imidazoline), 7.05–7.82 (*m*, 14H, Ar-H); Anal. Calcd. for C_25_H_18_ClN_5_ (423.13): C, 70.92; H, 4.26; N, 16.55%. Found: C, 70.78; H, 4.49; N, 16.30%.

***7–(3-Chlorophenyl)-8,9-diphenyl-3,7-dihydro-2H-imidazo[1,2-c]pyrrolo[3,2-e][1,2,3]triazine (4d):*** Yield: 78%; m.p.: 183–185 °C; IR (KBr) υ (cm^−1^): 1607 (C = N); MS (EI) *m*/*z*: 423 (M^+^, 30.17%); ^1^H-NMR (DMSO-d_6_, 300 MHz) δ (ppm): 2.53 (*t*, 2H, *J* = 7.4 Hz, CH_2_ imidazoline), 3.43 (*t*, 2H, *J* = 7.4 Hz, CH_2_ imidazoline), 6.82–7.65 (*m*, 14H, Ar-H); Anal. Calcd. for C_25_H_18_ClN_5_ (423.13): C, 70.92; H, 4.26; N, 16.55%. Found: C, 70.76; H, 4.11; N, 16.60%.

#### General procedure for the synthesis of compounds 5a-d:

A mixture of **2a-d** (5 mmol) and carbon disulphide (15 ml) in dry pyridine (50 ml) was heated under reflux for 10 h. The solid product formed on hot was filtered, washed with water, dried and crystallised from dioxane-water (2: 1) to afford **5a-d**

***7–(4-methoxyphenyl)-9-phenyl-6,7-dihydro-2H-imidazo[1,2-c]pyrrolo[3,2-e]pyrimidine-5(3H)-thione (5a):*** Yield: 70%; m.p.: 210–212 °C; IR (KBr) υ (cm^−1^): 3214 (NH), 1606 (C = N), 1490, 1260, 1025, 800 (C = S); MS (EI) *m*/*z*: 374 (M^+^, 39.2%); ^1^H-NMR (DMSO-d_6_, 300 MHz) δ (ppm): 3.32 (*t*, 2H, *J* = 7.2 Hz, CH_2_ imidazoline), 4.36 (*s*, 3H, OCH_3_), 4.56 (*t*, 2H, *J* = 7.2 Hz, CH_2_ imidazoline), 7.10–8.45 (*m*, 10H, Ar-H), 8.88 (*s*, 1H, NH D_2_O exchangeable); Anal. Calcd. for C_21_H_18_N_4_OS (374.12): C, 67.38; H, 4.81; N, 14.97%. Found: C, 67.09; H, 4.91; N, 15.13%.

***7–(4-methoxyphenyl)-8,9-diphenyl-6,7-dihydro-2H-imidazo[1,2-c]pyrrolo[3,2-e]pyrimidine-5(3H)-thione (5 b):*** Yield: 71%; m.p.: 187–189 °C; IR (KBr) υ (cm^−1^): 3307 (NH), 1586 (C = N), 1492, 1280, 1010, 800 (C = S); MS (EI) *m*/*z*: 450 (M^+^, 76.28%); ^1^H-NMR (DMSO-d_6_, 300 MHz) δ (ppm): 2.53 (*t*, 2H, *J* = 7.2 Hz, CH_2_ imidazoline), 2.65 (*t*, 2H, *J* = 7.2 Hz, CH_2_ imidazoline), 3.56 (*s*, 3H, OCH_3_), 7.18–7.93 (*m*, 14H, Ar-H), 8.69 (*s*, 1H, NH D_2_O exchangeable); Anal. Calcd. for C_27_H_22_N_4_OS (450.15): C, 72.0; H, 4.89; N, 12.44%. Found: C, 72.13; H, 5.08; N, 12.28%.

***7–(4-Chlorophenyl)-8,9-diphenyl-6,7-dihydro-2H-imidazo[1,2-c]pyrrolo[3,2-e]pyrimidine-5(3H)-thione (5c):*** Yield: 69%; m.p.: 138–140 °C; IR (KBr) υ (cm^−1^): 3214 (NH), 1606 (C = N), 1488, 1273, 1017, 805 (C = S); MS (EI) *m*/*z*: 454 (M^+^, 71.9%); ^1^H-NMR (DMSO-d_6_, 300 MHz) δ (ppm): 2.69 (*t*, 2H, *J* = 8.1 Hz, CH_2_ imidazoline), 3.43 (*t*, 2H, *J* = 8.1 Hz, CH_2_ imidazoline), 7.01–8.13 (*m*, 14H, Ar-H), 12.17 (*s*, 1H, NH D_2_O exchangeable); Anal. Calcd. for C_26_H_19_ClN_4_S (454.1): C, 68.72; H, 4.19; N, 12.33%. Found: C, 68.78; H, 4.41; N, 12.20%.

***7–(3-Chlorophenyl)-8,9-diphenyl-6,7-dihydro-2H-imidazo[1,2-c]pyrrolo[3,2-e]pyrimidine-5(3H)-thione (5d):*** Yield: 65%; m.p.: 172–174 °C; IR (KBr) υ (cm^−1^): 3309 (NH), 1585 (C = N), 1479, 1283, 1020, 798 (C = S); MS (EI) *m*/*z*: 454 (M^+^, 44.19%); ^1^H-NMR (DMSO-d_6_, 300 MHz) δ (ppm): 2.48 (*t*, 2H, *J* = 7.8 Hz, CH_2_ imidazoline), 3.32 (*t*, 2H, *J* = 7.8 Hz, CH_2_ imidazoline), 6.92–7.61 (*m*, 14H, Ar-H), 8.65 (*s*, 1H, NH D_2_O exchangeable); Anal. Calcd. for C_26_H_19_ClN_4_S (454.1): C, 68.72; H, 4.19; N, 12.33%. Found: C, 68.80; H, 4.21; N, 12.25%.

#### General procedure for the synthesis of compounds 6a-e:

A mixture of **5a-c** (1 mmol), the appropriate halo derivative (ethyl chloroformate, ethyl chloroacetate, phenacyl bromide) (1 mmol) and sodium acetate (0.6 g) in ethanol (40 ml) was heated under reflux for 3 h. The reaction mixture was then cooled and poured into water. The solid precipitate was filtered off, washed with water, dried and recrystallized from ethanol to give **6a-e**

***O-ethyl S-7–(4-methoxyphenyl)-9-phenyl-3,7-dihydro-2H-imidazo[1,2-c]pyrrolo[3,2-e] pyrimidin-5-yl carbonothioate (6a):*** Yield: 72%; m.p.: 180–182 °C; IR (KBr) υ (cm^−1^): 1765 (C = O), 1606 (C = N); MS (EI) *m*/*z*: 446 (M^+^, 20.2%); ^1^H-NMR (DMSO-d_6_, 300 MHz) δ (ppm): 1.21 (*t*, 3H, *J* = 7.5 Hz, CH_3_*-CH_2_), 2.50 (*t*, 2H, *J* = 7.8 Hz, CH_2_ imidazoline), 3.39 (*q*, 2H, *J* = 7.5 Hz, CH_2_*-CH_3_), 3.54 (*s*, 3H, OCH_3_), 3.77 (*t*, 2H, *J* = 7.8 Hz, CH_2_ imidazoline), 6.98–7.54 (*m*, 10H, Ar-H); Anal. Calcd. for C_24_H_22_N_4_O_3_S (446.14): C, 64.57; H, 4.93; N, 12.56%. Found: C, 64.29; H, 4.90; N, 12.88%.

***O-ethyl S-7–(4-methoxyphenyl)-8,9-diphenyl-3,7-dihydro-2H-imidazo[1,2-c]pyrrolo[3,2-e]pyrimidin-5-yl carbonothioate (6 b):*** Yield: 78%; m.p.: 163–165 °C; IR (KBr) υ (cm^−1^): 1720 (C = O), 1616 (C = N); MS (EI) *m*/*z*: 536 (M^+^, 13.42%); ^1^H-NMR (DMSO-d_6_, 300 MHz) δ (ppm): 1.24 (*t*, 3H, *J* = 7.4 Hz, H_3_*-CH_2_), 2.64 (*s*, 2H, CH_2_-S), 3.37 (*t*, 2H, *J* = 8.2 Hz, CH_2_ imidazoline), 3.96 (*q*, 2H, *J* = 7.4 Hz, CH_2_*-CH_3_), 4.32 (*s*, 3H, OCH_3_), 4.57 (*t*, 2H, *J* = 8.2 Hz, CH_2_ imidazoline), 7.01–8.86 (*m*, 14H, Ar-H); Anal. Calcd. for C_31_H_28_N_4_O_3_S (536.18): C, 69.40; H, 5.22; N, 10.45%. Found: C, 69.19; H, 5.40; N, 10.23%.

***2–(7-(4-chlorophenyl)-8,9-diphenyl-3,7-dihydro-2H-imidazo[1,2-c]pyrrolo[3,2-e]pyrimidin-5-ylthio)-1-phenylethanone (6c):*** Yield: 68%; m.p.: 165–167 °C; IR (KBr) υ (cm^−1^): 1734 (C = O), 1599 (C = N); MS (EI) *m*/*z*: 572 (M^+^, 18.9%); ^1^H-NMR (DMSO-d_6_, 300 MHz) δ (ppm): 2.92 (*s*, 2H, CH_2_-S), 4.16 (*t*, 2H, *J* = 7.5 Hz, CH_2_ imidazoline), 4.31 (*t*, 2H, *J* = 7.5 Hz, CH_2_ imidazoline), 6.69–7.43 (*m*, 19H, Ar-H); Anal. Calcd. for C_34_H_25_ClN_4_OS (572.14): C, 71.33; H, 4.37; N, 9.79%. Found: C, 71.57; H, 4.49; N, 9.52%.

***2–(7-(4-methoxyphenyl)-8,9-diphenyl-3,7-dihydro-2H-imidazo[1,2-c]pyrrolo[3,2-e]pyrimidin-5-ylthio)-1-phenylethanone (6d):*** Yield: 73%; m.p.: 150–152 °C; IR (KBr) υ (cm^−1^): 1719 (C = O), 1594 (C = N); MS (EI) *m*/*z*: 568 (M^+^, 65.38); ^1^H-NMR (DMSO-d_6_, 300 MHz) δ (ppm): 2.48 (*t*, 2H, *J* = 8 Hz, CH_2_ imidazoline), 3.35 (*s*, 3H, OCH_3_), 3.97 (*t*, 2H, *J* = 8 Hz, CH_2_ imidazoline), 4.08 (*s*, 2H, S-CH_2_-C = O), 6.72–7.60 (*m*, 19H, Ar-H); Anal. Calcd. for C_35_H_28_N_4_O_2_S (568.19): C, 73.94; H, 4.93; N, 9.86%. Found: C, 73.80; H, 5.02; N, 9.75%.

***2–(7-(4-methoxyphenyl)-9-phenyl-3,7-dihydro-2H-imidazo[1,2-c]pyrrolo[3,2-e]pyrimidin-5-ylthio)-1-phenylethanone (6e):*** Yield: 80%; m.p.: 171–173 °C; IR (KBr) υ (cm^−1^): 1721 (C = O), 1587 (C = N); MS (EI) *m*/*z*: 492 (M^+^, 17.33); ^1^H-NMR (DMSO-d_6_, 300 MHz) δ (ppm): 2.53 (*t*, 2H, *J* = 7.2 Hz, CH_2_ imidazoline), 3.35 (*s*, 3H, OCH_3_), 3.73 (*t*, 2H, *J* = 7.2 Hz, CH_2_ imidazoline), 4.09 (*s*, 2H, S-CH_2_-C = O), 6.580–7.51 (*m*, 15H, Ar-H); Anal. Calcd. for C_29_H_24_N_4_O_2_S (492.16): C, 70.73; H, 4.88; N, 11.38%. Found: C, 70.99; H, 4.70; N, 11.67%.

### 2.2. Biological method

#### 2.2.1. Microbial strains media, reagents and chemicals

Bacterial strains used were two strains of Gram-negative bacteria, *E. coli* O157:H7 ATCC 51659 and *P. aeruginosa* ATCC 27853 and two strains of Gram-positive bacteria: *S. aureus* ATCC9144 and *B. subtilis* ATCC 6051. One strain of unicellular fungi (yeast) was used: *Candida albicans* (*C. albicans*) ATCC 90028 and also two multicellular filamentous fungi: *F. oxysporum* and *A. fumigatus.*

Antibiotics were purchased commercially. All media were prepared and sterilised by autoclaving at 121 °C for 15 min. Nutrient agar (Merck, Germany), nutrient broth, Trypticase soy broth and agar (Biolife, Italy) were used for bacteria. Sabouraud dextrose agar (SDA) and SD broth were used for *C. albicans*. Potato dextrose agar (PDA) medium was used for the filamentous fungal species (*F. oxysporum* and *A. fumigatus*). The pH value of the PDA and SDA is 5.6 ± 0.2 at 25 °C. The pH values of the nutrient agar, broth, and trypticase soy broth media are (6.9, and 7.3 ± 0.2) respectively. All chemical compounds to be tested were dissolved in DMSO. The PH of the tested compounds was neutral.

#### 2.2.2. Determination of sensitivity of some Gram-positive, gram-negative bacteria and C. albicans to the synthesised compounds

The sensitivity was determined according to Kirby–Bauer disc diffusion method^52^^,^^53^. Suspensions of bacterial and fungal standard strains were prepared from fresh slants in 3 ml sterile saline solution and the suspensions were adjusted to 0.5 McFarland (1–2 × 10^8^ cfu/mL) then inoculated (5 drops) uniformly in nutrient agar plates for bacteria and SDA plates for *C. albicans*. Dried filter paper discs loaded with a standard amount (1000–2000 *µ*g/mL) of the test compounds were applied to the surface of the plate. The plates were incubated at (37 °C for 24 h for bacteria) and (28 °C for 48 h for fungi) and sensitivity was determined by presence of zones of inhibition of microbial growth around the test compound disc. DMSO-loaded disc was used as a negative control. Ciprofloxacin disc was used as a standard antibacterial drug, and amphotericin B disc was used as a standard antifungal one. All experiments were conducted in triplicate and the mean values of the inhibition zones were calculated.

#### 2.2.3. Determination of minimum inhibitory concentration (MIC) of the synthesised compounds against some Gram-positive, Gram-negative bacteria and C. albicans

The MICs of the tested compounds and control drugs against relevant bacterial and fungal strains were determined using the broth macrodilution method, according to guidelines outlined by the Clinical and Laboratory Standards Institute^53–55^.

Bacterial strains were grown aerobically overnight on nutrient agar at 37 °C, *C. albicans* was grown aerobically for 48 h. on SDA at 35 °C. Afterwards, suspensions of bacterial and fungal standard strains were prepared in 3 ml sterile saline solution and adjusted to 0.5 McFarland (1–2 × 10^8^ cfu/mL). Tested compounds used to prepare stock solutions in DMSO at concentration of (1000 *µ*g/mL) each. Further, test compounds were serially diluted in the corresponding culture medium (Trypticase soy broth and nutrient broth for antibacterial and SD broth for antifungal) in test tubes to achieve concentrations ranging from 50 to 400 *µ*g/mL. Finally, the different strains were inoculated at 5 × 10^5^ cfu/mL final concentration. The tubes were incubated aerobically at (37 °C for 24 h for bacteria) and (28 °C for 48 h for fungi) and the MICs were determined by visually assessing turbidity after the specified time. MIC was defined as the lowest concentration that resulted in complete lack of turbidity after the specified incubation period was conducted. For each tested compound, triplicate measurements were conducted. The mean MIC values, standard deviation (SD) and statistical differences were calculated using GraphPad Prism version 5 for Windows (GraphPad Inc., USA). DMSO was used as a negative control. **Ciprofloxacin** was used as standard antibacterial drug in bacteria experiments and **amphotericin B** was used as a standard antifungal one.

#### 2.2.4. Molecular identification of the filamentous fungal species

##### 2.2.4.1 DNA isolation of the filamentous fungal species

Molecular identification was carried out on the two filamentous fungal species used in this study. In summary, the genomic DNA was isolated by using a mortar and pestle to grind approximately 100 mg of frozen fungal mycelia with liquid nitrogen and mixing with 1 ml of 4 M guanidinium thiocyanate, 0.1 M sodium acetate pH 5.5, 10 mM ethylene-di-amine-tetra acetic acid (EDTA), 0.1 M 2-mercaptoethanol. Centrifugation of the extracts and the supernatants were loaded into a silica gel spin column (Wizard Plus SV Minipreps DNA Purification, Promega, USA). Finally, columns were washed with 70% ethanol, 10 mM sodium acetate pH 5.5 and DNA eluted with 50 µL of 20 mM Tris–HCl, pH 8.5.

##### 2.2.4.2 Ribosomal DNA amplification and sequencing of the filamentous fungal species

PCR was carried out to amplify the ribosomal internal transcribed sequence (ITS) using primer pairs ITS1 (5′-CTTGGTC ATTTAGAGGAAGTAA-3′) and ITS4 (5′-TCCTCCGC TTATTGATATGC-3′)^56^. Fungal DNA (1 µL) was amplified in a 20 µL reaction with 0.4 U Phusion DNA polymerase (Thermo Scientific) in the presence of 0.2 mM dNTPs, 3% dimethyl sulfoxide, 0.5 µM each primer and HF buffer (Thermo Scientific). Reaction consisted of an initial denaturation for 30 s at 98 °C, followed by 30 cycles of 10 s at 98 °C, 30 s at 55 °C and 30 s at 72 °C, and a final extension of 10 min at 72 °C. PCR products were separated by electrophoresis in a 1% agarose gel run for 75 min in buffer TAE (40 mM Tris, 20 mM sodium acetate, 1 mM EDTA, pH 7.2) and visualised using a UV transilluminator after ethidium bromide staining. The PCR products corresponding to ribosomal ITS, according to electrophoretic migration, were eluted from the gel using silica spin columns (DNA Clean and Concentrator, Zymo Research). Both strands of amplified ribosomal ITS DNA were sequenced using primers ITS1 and ITS4. The consensus sequence was used to search for homologous sequences using the BLAST search program at National Centre for Biotechnology Information (NCBI; http://www.ncbi.nlm.nih.gov).

#### 2.2.5. In vitro antifungal activity of the synthetic compounds against two filamentous fungal species

Poisoned food technique^57^ was used to measure the antifungal activity of the newly synthesised compounds on two different pathogenic filamentous fungal species (*F. oxysporum* and *A. fumigatus)* that cause human and plant diseases as well. In summary, three different concentrations (1, 10, 50) µg/mL for each compound were used separately to supplement the agar medium, then from each of fungal species a (0.5 cm) disc of the active mycelial growth, eight-day-old fungal culture, was placed in the centre of each prepared plate. Finally, plates were incubated at 26 °C ± 2 for 8 days. The antifungal effect of synthetic compound was evaluated by measuring the radial growth of fungal growth in each inoculated plate^58^ and calculating the inhibitory growth rate:

Inhibitory growth rate (%) = (R−r)/R x 100

“where **R** is the radial growth of fungal hyphae on the control plate and **r** is the radial growth of fungal hyphae on the plate supplemented with the synthetic compound”. **Itraconazole** (Johnson & Johnson, New Jersey, USA) and **Kocide 2000** (Certis USA, Columbia, MD) were used as positive control. The experiments were repeated three times. The mean values of Inhibitory growth rate (%), standard deviation (SD) and statistical differences were calculated using GraphPad Prism version 5 for Windows (GraphPad Inc., USA).

#### 2.2.6. The effect of the tested compounds on some selected species nucleic acid

##### 2.2.6.1 The effect on the bacterial nucleic acid

DNA damage analysis was performed on *P. aeruginosa* ATCC 27853 according to^59^ as a representative of the bacterial strains used in this study. Total DNA was isolated from *P. aeruginosa* cells after three hours treatment with each the MIC value of each tested compound individually. Isolation of *P. aeruginosa* DNA and polymerase chain reaction (PCR) amplification of the 16S–23SrDNA intergenic transcribed spacer Isolated from each microcosm were cultivated on LB agar for 24 h at 30 W C, then the total nucleic acids were extracted by means of the InstaGene™ Matrix (bio-Rad Laboratories) according to the protocol described by the manufacturer. The corresponding DNA encoding 16S–23S rDNA intergenic transcribed spacer (ITS) was amplified from genomic DNA using forward primer Paer1, 5′-TCCAAA CAATCGAAAGC-3′ and reverse primer Paer2, 5′- CCGAAAACTTGCGCTTGAAC-3′. All of the amplifications were performed in a 100 PTC- thermal cycler, as previously reported (Tyler *et al.* 1995)^60^. The reaction mixture (50 μL) included 1x reaction buffer (5 μL), 0.2 mM of each deoxynucleoside triphosphate, 1 μM of forward and reverse primer, 1 μL of the DNA template, 1.5 mM of MgCl_2_, and 2.5 U of Taq DNA polymerase. Each PCR reaction consisted of 30 amplification cycles, with an initial denaturation of 2 min at 94 °C followed successively by denaturation (30 s) at 94 °C, annealing (30 s) at 60 °C, and extension (1 min), ending with a 10 min extension at 72 °C and subsequent cooling to 4 °C. The molecular weight standard used was DNA Hyper Ladder II (Promega, USA). The PCR products were loaded onto a 1% agarose gel for electrophoresis followed by ethidium bromide staining^61^.

##### 2.2.6.2 The effect on the filamentous fungal nucleic acid

DNA damage study on *F. oxysporum* was performed according to the protocol of^58^^,^^62^^,^^63^. DNA amplified by PCR was treated with our synthetic compounds at (1 mM) and incubated for 1 h. DNA without the compound treatment served as a control. After treatment DNA was separated by electrophoresis using a 1% agarose gel at 75 V for 30 min. The gel was stained with ethidium bromide and then subjected to UV irradiation to visualise the DNA bands.

## 3. Results and discussion

### 3.1. Chemistry

*N*-aryl-2-amino-3-cyanopyrroles **1a-d** were obtained by reaction of α-hydroxy ketones (namely; benzoin or phenacyl bromide) with different aryl amine and malonodinitrile according to a procedure defined in the literature^50^^,^^64^^,^^65^. Reaction of nitriles, carboxylic acids, esters, ortho-esters or hydroxyamides with ethylenediamine, using carbon disulphide as solvent, is a well-known method for preparation of the corresponding 2-substituted imidazolines, as used here for the preparation of compounds 3–(2-dihydroimidazolyl)pyrrole-2-amines **2a-d**^66–69^. The structures of these compounds were confirmed by spectral analysis; IR spectra showed disappearance of CN absorption bands and appearance of absorption frequency for NH at 3403–3290 cm^−1^. ^1^H-NMR spectra revealed the appearance of two triplet peaks for two methylene groups of imidazoline ring in the range 2.53–4.57 ppm and distinctive D_2_O exchangeable peak of NH at 8.84–9.64 ppm.

On the other hand, the 3–(2-dihydroimidazolyl)pyrrole-2-amines (**2a-d)** were converted to the corresponding pyrrolo[3,2*-e*]imidazo[1,2*-c*]pyrimidines (**3a-d, 5a-d)** *via* condensation with triethylorthoformate (TEOF) or carbon disulphide (CS_2_), respectively^70–72^. Structures of pyrrolo[3,2-e]imidazo[1,2-c]pyrimidine **3a-d** were confirmed by disappearance of both NH and NH_2_ absorption bands in IR spectra and also their peaks in^1^H-NMR spectra were disappeared and in Mass spectra molecular ion peaks were recorded exactly at the molecular weight of each compound. Also structures of pyrrolo[3,2-*e*]imidazo[1,2-*c*]pyrimidine-5-thiones **5a-d** were confirmed by disappearance of NH_2_ absorption bands in IR spectra, but NH absorption bands were still present, and appearance of four distinctive absorption bands for C = S group at 1492–798 cm^−1^. ^1^H-NMR spectra also revealed disappearance of NH_2_ peaks and presence of NH peaks.

Formation of pyrrolo[3,2-*e*]imidazo[1,2-*c*]1,2,3-triazines **(4a-d)** were obtained *via* reaction of **2 a-d** with sodium nitrite (NaNO_2_)^73–75^, Confirmation of structures of compounds **4a-d** was lied on disappearance of both NH and NH_2_ absorption bands or peaks in both IR and ^1^H-NMR spectra. Alkylation of thione derivatives **5a-d** with appropriate halo derivative (namely; ethyl chloroformate, ethyl chloroacetate, or phenacyl bromide) afforded alkylated thio derivatives **6a-e.** The structures of these compounds were confirmed by spectral analysis; IR spectra showed disappearance of both NH and C = S absorption bands and appearance of absorption frequency for C = O at 1765–1719 cm^−1^. ^1^H-NMR spectra revealed the disappearance of NH peaks and appearance of new peaks in a reasonable correspondence with excess aliphatic part in the compounds. The mass spectra showed molecular ion peaks at *m/z* values as it should be for each derivative, as revealed in Scheme 1.

### 3.2. Anti-microbial activities

#### 3.2.1. Sensitivity of some gram-positive, gram-negative bacteria and C. albicans to the tested compounds:

The newly synthesised compounds were tested for antimicrobial activity by the disc diffusion method. Results are shown in Table 1.

**Table 1.** The *in vitro* antimicrobial activity of the synthesised compounds and the control drugs (1000 *µ*g/mL) against some Gram-positive, Gram-negative bacteria and *C. albicans.*

Microorganisms, Mean zone of inhibition (MZI) (diameter, in mm) ± SD					
Tested Compound	Gram + ve		Gram –ve		
	*Staphylococcus aureus* (ATCC9144)	*Bacillus subtilis* (ATCC 6051)	*Escherichia coli* O157:H7 (ATCC 51659)	*Pesudomonas aeruginosa* (ATCC 27853)	*Candida albicans* (ATCC 90028)
**2a**	22.00 ± 0.50^ca^	24.00 ± 0.35^ca^	16.00 ± 1.5^c^	18.00 ± 2.00^c^	24.00 ± 2.00^cb^
**3a**	15.00 ± 1.00^c^	17.00 ± 1.75^c^	22.00 ± 0.85^c^	23.00 ± 3.00^c^	18.00 ± 1.00^c^
**3b**	24.00 ± 1.25^ca^	21.00 ± 0.25^ca^	19.00 ± 0.65^c^	19.00 ± 2.00^c^	24.00 ± 1.00^cb^
**3c**	25.00 ± 1.00^ca^	24.00 ± 1.00^ca^	19.00 ± 1.25^c^	16.00 ± 3.00^c^	23.00 ± 3.00^cb^
**3d**	21.00 ± 2.00^c^	20.00 ± 0.2.00^ca^	22.00 ± 0.55^c^	18.00 ± 3.00^c^	19.00 ± 1.00^c^
**4a**	20.00 ± 0.30^c^	18.00 ± 2.00^c^	21.00 ± 0.15^c^	22.00 ± 4.00^c^	18.00 ± 1.00^c^
**4d**	21.00 ± 1.00^ca^	23.00 ± 2.00^ca^	20.00 ± 0.50^c^	21.00 ± 1.00^c^	28.00 ± 1.00^cb^
**5a**	17.00 ± 0.75^c^	21.00 ± 1.00^ca^	22.00 ± 0.35^c^	24.00 ± 1.00^c^	21.00 ± 0.55^c^
**5b**	20.00 ± 0.50^c^	22.00 ± 3.00^ca^	23.00 ± 1.85^c^	21.00 ± 1.00^c^	22.00 ± 2.00^cb^
**5c**	21.00 ± 0.75^ca^	22.00 ± 0.35^ca^	24.00 ± 2.00^c^	23.00 ± 3.00^c^	19.00 ± 2.00^c^
**5d**	18.00 ± 2.00^c^	19.00 ± 0.55^c^	20.00 ± 0.15^c^	20.00 ± 3.00^c^	22.00 ± 0.25^cb^
**6a**	17.00 ± 0.25^c^	18.00 ± 0.85^c^	^c^19.00 ± 2.00^c^	21.00 ± 1.00^c^	18.00 ± 1.00^c^
**6b**	18.00 ± 0.50^c^	17.00 ± 2.00^c^	21.00 ± 0.75^c^	22.00 ± 2.00^c^	22.00 ± 0.65^cb^
**6c**	15.00 ± 2.00^c^	21.00 ± 1.00^ca^	18.00 ± 1.00^c^	18.00 ± 2.00^c^	9.00 ± 0.25^c^
**6d**	19.00 ± 2.00^c^	19.00 ± 0.55^c^	22.00 ± 2.00^c^	20.00 ± 3.00^c^	10.00 ± 0.85^c^
**6e**	21.00 ± 2.00^ca^	22.00 ± 0.50^ca^	16.00 ± 1.00^c^	18.00 ± 3.00^c^	24.00 ± 0.75^cb^
**Amphotericin B**	NT	NT	NT	NT	22.00 ± 0.50^c^
**Ciprofloxacin**	19.00 ± 2.00^c^	18.00 ± 0.95^c^	26.00 ± 1.00^c^	25.00 ± 3.00^c^	NT
**DMSO**	Nil	Nil	Nil	Nil	Nil

Data shown in Table 1 are expressed as mean ± SD (Standard deviation);.

"Nil "indicates no significant inhibitory effect (<5 mm) and "NT" indicates Not tested.

^a^Significant from (Ciprofloxacin) positive control for bacteria at *p* < 0.05.

^b^Significant from (Amphotericin B) positive control for *C. albicans* at *p* < 0.05.

^c^Significant from (DMSO) negative control at *p* < 0.05.

***To discuss the former results***, the results indicated good antimicrobial activities for all tested compounds. Among these, compounds **2a**, **3c** and **4d** were found to possess significant activity against Gram-positive bacteria and *C. albicans* when compared to standard antibacterial and antifungal drugs with compound **3c** as the most potent one. Their mean zones inhibition (MZI) were: **2a** (22, 24, and 24 mm), **3c** with the highest MZI (25, 24, and 23 mm), **4d** (21, 23, and 28 mm) against *S. aureus, B. subtilis* and *C. albicans,* respectively. Where **2a**, **3c** and **4d** shown higher activity over standard drug **Ciprofloxacin** (19 and 18 mm) *against S. aureus,* and *B. subtilis.* All the three compounds **2a, 3c and 4d** gave better anticandidal effect with higher MZI values than the standard drug **Amphotericin B** (22 mm) and the most potent was 4d. For *E. coli,* the best effective compounds were **5 b** and **5c** with MZI (23 and 24 mm), respectively. While for *P. aeruginosa,* the best effective compounds were **5a** and **5c** with MZI (24 and 23 mm), respectively.

#### 3.2.2. MIC Of the synthesised compounds against some Gram-positive, Gram-negative bacteria and C. albicans

To further evaluate antimicrobial potential, the compounds that gave the highest MZI against the tested microorganisms were selected to get their MIC determined against the corresponding genus. Results are shown in Table 2.

**Table 2.** The MIC (µg/mL) of the synthesised compounds and a control drugs using the broth macrodilution method.

Tested Compound/ Control Antibiotics	Microorganisms, MIC (µg/mL) ± SD				
	Gram-positive		Gram-negative bacteria		***C. albicans*** **ATCC 90028**
	*S. aureus* ATCC9144	***B. subtilis*** **ATCC 6051**	***E. coli* O157:H7** **ATCC 51659**	***P. aeruginosa*** **ATCC 27853**	
**2a**	**30.00 ± 1.15**	**33.00 ± 2.00**	NT	NT	**15.00 ± 1.75**
**3c**	**30.00 ± 0.75**	**31.00 ± 0.95**	NT	NT	**30.00 ± 1.50**
**4d**	**35.00 ± 0.50**	**33.00 ± 0.85**	NT	NT	**12.00 ± 1.65**
**5a**	NT	NT	NT	**40.00 ± 0.50**	NT
**5b**	NT	NT	**25.00 ± 0.65**	NT	NT
**5c**	NT	NT	**20.00 ± 0.85**	**30.00 ± 1.00**	NT
**Amphotericin B**	NT	NT	NT	NT	**56.00 ± 1.00**
**Ciprofloxacin**	**45.00 ± 0.65**	**40.00 ± 1.50**	**25.00 ± 1.25**	**30.00 ± 1.25**	NT
**DMSO**	Nil	Nil	Nil	Nil	Nil

Data shown in Table 2 are expressed as mean ± SD (standard deviation) of triplicates.

"Nil "indicates no inhibitory effect (turbid test tube) and "NT" indicates Not tested.

Results showed that all the tested compounds against both *S. aureus* and *B. subtilis* (**2a, 3c** and **4d**) showed a higher antibacterial effect than the standard drug, with their MIC values (30, 30, 35 µg/mL), respectively against *S. aureus* and (33, 31, 33 µg/mL), respectively, against *B. subtilis*, all of them are less than the MIC values of ciprofloxacin (45 and 40 µg/mL) against *S. aureus* and *B. subtilis,* respectively. Compound **3c** is considered the best antimicrobial compound against both of the tested Gram-positive bacteria with a slight difference from compounds **2a** and **4d**. As for *E. coli,* **5 b** has an MIC (25 µg/mL) that’s equal to ciprofloxacin (25 µg/mL) **5c** has almost a lower one (20 µg/mL), which means better antibacterial effect. Compound **5c** is considered to have the best antimicrobial effect against both Gram-negative bacterial strains because with *P. aeruginosa,* it had the same MIC value as ciprofloxacin (30 µg/mL), while compound **5a** had a higher one (40 µg/mL).

***From the former results,*** the relatively low effect of the tested compounds on *P. aeruginosa* in comparison to their effect on the other tested strains (Table 2) is due to the fact that it is remarkably capable of resisting antibiotics and its strains have high levels of both intrinsic and acquired resistance mechanisms to most antibiotics. Moreover, *P. aeruginosa* has adaptive antibiotic resistance mechanisms like biofilm- mediated resistance and formation of multidrug-tolerance^3^. It can also be noticed from the results (Tables 1 and 2) that the antibacterial activity of the tested compounds against Gram-positive strains in general was higher than that against Gram-negative ones. This result can be explained by the fact that Gram-negative bacteria are more resistant than Gram-positive ones because of their special structure^76^ that differs in the composition of the cell wall^77^^,^^78^. Gram-negative bacteria have an outer membrane (OM), while Gram-positive bacteria do not and the OM of former is responsible for making them resistant to many antibiotics, because most antibiotics have to pass through it to enter the cell^79^.

As for the fungus *C. albicans*; all the tested compounds possessed very significant antifungal activities that are much higher than the standard drugs as the MIC values of compounds (**2a, 3c** and **4d**) were (15, 30 and 12 µg/mL), respectively, while that of **amphotericin B** was (56 µg/mL). Compound **4d** had the best antifungal effect with an MIC that’s almost fivefold lower than that of **amphotericin B**, with a slight difference from compound **2a**, MIC almost four-fold the **amphotericin B** one. The results indicated that compounds **2a, 3c** and **4d** have a very good activity against both Gram-positive bacteria and candida but that the anti-candida effect is higher than the antibacterial one, these compounds exceeded one of the most active antifungal agent, **amphotericin B,** against most Candida species^6^.

#### 3.2.3. Identification of the filamentous fungal species

Accurate identification of both used species was confirmed by sequence analysis of the nuclear ribosomal ITS region as *F. oxysporum* and *A. fumigatus.*

#### 3.2.4. In vitro antifungal activity of the synthetic compounds against two filamentous fungal species

We used the poison food technique to analyse the *in vitro* inhibitory effect of the tested compounds at different concentrations (1, 10 and 50) µg/mL compared to two chemical fungicides: **Kocide 2000** 2500 µg/mL and **itraconazole** 6.25 µg/mL. Results are shown in Table 3 and 4.

**Table 3.** Mean of inhibitory growth rate (%) ±SD of a pathogenic *F. oxysporum* with the tested compounds: All control plates have 100 ± 0 growth rate on the 8^th^ day of cultivation.

Tested Compounds/ Control antifungal	**Concentration** **µg/mL**	Inhibition rate (%)± SD		
		4 days	6 days	8 days
2a	1	40.12 ± 1.70	42.13 ± 2.1	42.12 ± 4.1^#^
	10	42.13 ± 1.03	42.01 ± 1.2	42.65 ± 1.2
	50	43.55 ± 1.5	43.10 ± 1.2	44.2 ± 2.1*
3a	1	80.12 ± 1.33	80.12 ± 2.2	81.29 ± 1.9^#^
	10	81.22 ± 1.20	82.33 ± 2.5	83.12 ± 2.5
	50	83.21 ± 1.7	83.77 ± 1.2	84.0 ± 1.9 *
3b	1	79.15 ± 2.30	80.24 ± 3.1	82.34 ± 0.5#
	10	80.93 ± 2.49	82.07 ± 2.5	84.13 ± 1.5
	50	82.72 ± 2.8	87.77 ± 2.8	88.8 ± 2.5*
3c	1	81.25 ± 2.30	82.25 ± 7.1	83.34 ± 2.5^#^
	10	82.83 ± 2.49	83.07 ± 1.8	85.13 ± 2.5
	50	86.72 ± 2.8	87.95 ± 2.8	89.9 ± 1.5*
3d	1	70.12 ± 2.23	70.11 ± 3.1	71.12 ± 1.5^#^
	10	71.15 ± 1.5	71.12 ± 2.1	72.14 ± 1.5
	50	73.12 ± 1.5	73.00 ± 1.1	73.22 ± 1.5
4a	1	72.12 ± 1.62	72.21 ± 3.2	72.10 ± 2.4^#^
	10	72.16 ± 2.31	72.21 ± 1.4	72.33 ± 2.5
	50	73.13 ± 2.5	73.12 ± 3.0	73.0 ± 1.0
4d	1	65.35 ± 1.5	73.0.5 ± 1.09	78.37 ± 2.1^#^
	10	71.21 ± 1.5	74.153 ± 1.09	78.5 ± 0.33
	50	76.95 ± 2.1	78.17 ± 1.08	80.12 ± 0.5*
5a	1	85.12 ± 3.20	86.52 ± 2.1	88.21 ± 0.4
	10	87.52 ± 2.5	87.90 ± 2.5	88.12 ± 1.6
	50	88.30 ± 1.5	88.10 ± 1.2	88.22 ± 7.0*
5b	1	81.15 ± 6.30	82.24 ± 7.1	83.34 ± 2.4^#^
	10	81.93 ± 2.49	82.07 ± 1.8	84.13 ± 2.5
	50	87.72 ± 2.8	87.77 ± 2.8	88.8 ± 2.5*
5c	1	34.21 ± 2.47	35.00 ± 2.12	36.52 ± 3.24^#^
	10	34.76 ± 1.3	35.13 ± 2.3	35.7 ± 1.77
	50	36.89 ± 1.26	37.17 ± 1.04	38.22 ± 1.5*
5d	1	71.15 ± 2.22	72.25 ± 2.2	74.37 ± 1.45^#^
	10	72.12 ± 2.1	73.53 ± 1.24	75.9 ± 2.33
	50	73.12 ± 2.11	74.22 ± 2.15	76.12 ± 2.5
6a	1	66.21 ± 1.2	72.0.5 ± 1.09	77.37 ± 2.1^#^
	10	70.1.08 ± 0.5	73.153 ± 1.09	77.5 ± 0.33
	50	75.89 ± 2.1	77.17 ± 1.08	79.12 ± 0.5*
6b	1	80.13 ± 2.30	81.20 ± 2.5	82.31 ± 2.0^#^
	10	81.23 ± 2.43	82.50 ± 1.8	84.15 ± 1.5
	50	85.72 ± 2.8	85.77 ± 2.8	88.8 ± 1.5*
6c	1	40.95 ± 1.0	40.22 ± 1.1	41.09 ± 1.5^#^
	10	41.21 ± 1.2	41.40 ± 2.3	42.12 ± 1.5
	50	42.32 ± 2.1	43.02 ± 2.2	44.0 ± 2.1*
6d	1	72.13 ± 1.55	72.33 ± 1.5	73.55 ± 1.5^#^
	10	73.11 ± 2.14	73.26 ± 1.7	74.12 ± 4.5
	50	74.19 ± 2.4	74.99 ± 7.3	75.2 ± 2.5
6e	1	80.15 ± 4.30	81.24 ± 7.1	82.34 ± 2.4^#^
	10	81.93 ± 2.49	81.07 ± 1.8	83.13 ± 1.5
	50	85.22 ± 2.5	85.72 ± 2.8	86.8 ± 2.5*
**Kocide 2000**	2500	64.5 ± 1.5	65.2 ± 0.4	70.5 ± 0.5
**Itraconazole**	6.25	82.0 ± 1.5	89.0 ± 0.6	93.5 ± 0.5

Data shown in Table 3 are expressed as mean ± SD (Standard deviation);.

*Significant difference from positive control (Kocide 2000 compared to concentration of 50 µg/mL of the tested compounds) at *p* < 0.05.

^#^Significant difference from positive control (Itraconazole compared to concentration of 1 µg/mL of the tested compounds) at *p* < 0.05.

**Table 4.** Mean of inhibitory growth rate (%) ±SD of a pathogenic *A. fumigatus* with tested compounds: All control plates have 100 ± 0 growth rate on the 8^th^ day of cultivation.

Tested Compounds/ Control antifungal	**Concentration** **µg/mL**	Inhibition rate (%)± SD		
		4 days	6 days	8 days
2a	1	40.12 ± 1.70	42.13 ± 2.1	42.12 ± 4.1^#^
	10	42.13 ± 1.03	42.01 ± 1.2	42.65 ± 1.2
	50	43.55 ± 1.5	43.10 ± 1.2	44.2 ± 2.1*
3a	1	80.12 ± 1.33	80.12 ± 2.2	81.29 ± 1.9^#^
	10	81.22 ± 1.20	82.33 ± 2.5	83.12 ± 2.5
	50	83.21 ± 1.7	83.77 ± 1.2	84.0 ± 1.9*
3b	1	79.32 ± 2.10	81.24 ± 3.5	83.34 ± 0.5^#^
	10	80.23 ± 2.49	81.07 ± 2.1	85.13 ± 1.5
	50	82.72 ± 2.8	85.77 ± 2.2	87.8 ± 2.5*
3c	1	81.25 ± 2.30	82.25 ± 7.1	83.34 ± 2.5^#^
	10	82.83 ± 2.49	83.07 ± 1.8	85.13 ± 2.5
	50	86.72 ± 2.8	87.95 ± 2.8	89.9 ± 1.5*
3d	1	70.12 ± 2.23	70.11 ± 3.1	71.12 ± 1.5^#^
	10	71.15 ± 1.5	71.12 ± 2.1	72.14 ± 1.5
	50	73.12 ± 1.5	73.00 ± 1.1	73.22 ± 1.5
4a	1	72.12 ± 1.62	72.21 ± 3.2	72.10 ± 2.4^#^
	10	72.16 ± 2.31	72.21 ± 1.4	72.33 ± 2.5
	50	73.13 ± 2.5	73.12 ± 3.0	73.0 ± 1.0
4d	1	65.35 ± 1.5	73.0.5 ± 1.09	78.17 ± 2.1^#^
	10	71.21 ± 1.5	74.153 ± 1.09	78.5 ± 0.33
	50	76.95 ± 2.1	78.17 ± 1.08	80.12 ± 1.5*
5a	1	85.12 ± 3.20	86.52 ± 2.1	88.21 ± 0.4
	10	87.52 ± 2.5	87.90 ± 2.5	88.12 ± 1.6
	50	88.30 ± 1.5	88.10 ± 1.2	88.22 ± 7.0*
5b	1	80.15 ± 2.3	81.34 ± 7.1	83.34 ± 2.4^#^
	10	81.43 ± 1.2	81.07 ± 1.8	84.22 ± 2.5
	50	86.32 ± 1.5	86.77 ± 2.8	88.21 ± 2.5*
5c	1	44.21 ± 2.54	45.00 ± 2.22	46.52 ± 3.62^#^
	10	44.77 ± 2.3	45.13 ± 2.3	47.7 ± 1.77
	50	46.89 ± 1.33	47.17 ± 2.04	48.22 ± 2.5*
5d	1	70.15 ± 3.2	71.25 ± 2.2	73.37 ± 1.45^#^
	10	72.12 ± 3.1	73.53 ± 2.5	74.9 ± 1.23
	50	73.12 ± 3.5	74.22 ± 4.2	75.12 ± 1.5
6a	1	65.34 ± 1.2	71.0.5 ± 2.25	76.54 ± 3.2^#^
	10	69.1.22 ± 0.5	71.26 ± 1.09	77.5 ± 2.22
	50	75.23 ± 2.2	71.24 ± 1.08	79.32 ± 0.5
6b	1	80.13 ± 2.30	81.20 ± 2.5	82.31 ± 2.0^#^
	10	81.23 ± 2.43	82.50 ± 1.8	84.15 ± 1.5
	50	85.72 ± 2.8	85.77 ± 2.8	88.8 ± 1.5*
6c	1	40.95 ± 1.0	40.22 ± 1.1	41.09 ± 1.5^#^
	10	41.21 ± 1.2	41.40 ± 2.3	42.12 ± 1.5
	50	42.32 ± 2.1	43.02 ± 2.2	44.0 ± 2.1*
6d	1	72.13 ± 1.55	72.33 ± 1.5	73.55 ± 1.5^#^
	10	73.11 ± 2.14	73.26 ± 1.7	74.12 ± 4.5
	50	74.19 ± 2.4	74.99 ± 7.3	75.2 ± 2.5
6e	1	80.15 ± 4.2	80.24 ± 7.1	82.22 ± 2.4^#^
	10	81.93 ± 2.1	81.07 ± 1.3	82.44 ± 1.5
	50	85.22 ± 2.3	84.72 ± 2.4	85.8 ± 2.5*
**Kocide 2000**	2500	60.4 ± 0.5	65.5 ± 1.5	72.5 ± 1.0
**Itraconazole**	6.25	82.0 ± 1.0	84.0 ± 0.5	92.2 ± 5.0

Data shown in Table 4 are expressed as mean ± SD (Standard deviation).

*Significant difference from positive control (Kocide 2000 compared to concentration of 50 µg/mL of the tested compounds) at *p* < 0.05.

^#^Significant difference from positive control (Itraconazole compared to concentration of 1 µg/mL of the tested compounds) at *p* < 0.05.

First: in comparison to **Kocide 2000 (**Cu(OH)_2_) that is used against plant pathogens, the obtained results clearly indicated that a number of our synthetic compounds markedly exhibited higher antifungal activity than the control drug. The mycelial growth of pathogenic *F. oxysporum* and *A. fumigatus* was inhibited to various extents our newly synthesised compounds.

A concentration of 50 µg/mL of the compounds **3a-d, 4a,d, 5a,b,d and 6a,b,d,e** induced the highest levels of inhibition rate (**%**) of mycelial growth of both of the tested pathogenic species *F. oxysporum* and *A. fumigatus* compared to the chemical fungicide (**Kocide 2000**) after incubation period of 8 days. The values against *F. oxysporum* were in range between (**73.0 ± 1.0 to 89.9 ± 1.5)** exceeded these of the chemical fungicide **Kocide 2000** (**70.5 ± 0.5**). On testing the other strain, *A. fumigatus,* the values of the chemical compounds (**3a-d, 4a,d, 5a,b,d and 6a,b,d,e**) were in range between (**73.0 ± 1.0 to 89.9 ± 1.5)** compared to the chemical fungicide value (**72.5 ± 1.0 Kocide 2000**). The compound **3c** is showing the highest inhibition rate (**%**) of the mycelial growth of both pathogenic species (*F. oxysporum* and *A. fumigatus*) with the value (**89.9 ± 1.5**).

***To Discuss the former results;*** The antifungal effect shown by the tested compounds was not only higher than that of the commercially well-known fungicide **Kocide 2000**, but also at a lower concentration than its recommended concentration (**2500 **µg/mL). That is why trying to find new antifungal agents that can be used on plants is a major concern. So that finding makes the synthesised compounds very promising candidates for commercial use as they produce the desired antifungal effect at a much lower concentration that can produce a safer alternative on edible plants but this needs further investigation. This result is highly important given the fact that *F. oxysporum* causes a global economic loss by infecting important plants^80^.

Second: in comparison to **itraconazole** (triazole derivative) as the control drug for treatment of the human mycelial diseases, the results (Table 3) showed that some compounds had a very good effect at different concentrations compared to itraconazole at **6.25 **µg/mL. The compounds **3 b, 3c, 5a, 5 b, 6 b and 6e** displayed good antifungal activity against the tested pathogenic species *F. oxysporum* and *A. fumigatus*. The values of mycelial growth inhibition rate (**%**) at a concentration of 1 µg/mL against both *F. oxysporum* and against *A. fumigatus* were in range between (**82.31 ± 2.0 to 88.21 ± 0.4**), in comparison to itraconazole at a concentration of (**6.25 **µg/mL) the values were (**93.5 ± 0.5 and 92.2 ± 5.0**), respectively. Compound **5a** had the highest activity with growth inhibition rate **%** (**88.21 ± 0.4**) at **1 **µg/mL against both *F. oxysporum* and *A. fumigatus*.

***To understand the former results;*** this result is very promising as it means that the antifungal effect of our tested compounds against the filamentous fungal human pathogens was highly equivalent to that of itraconazole and can be produced at even a lower concentration (1 µg/mL). Knowing that itraconazole is considered as one of the very few fungicides for human use that still can overcome the resistance of both of the species used in this study. If we look at the current treatments of A*. fumigatus* we can find **Amphotericin B** as an effective antifungal, yet with significant side effects. Other effective drugs are **echinocandins** (cyclic hexapeptide antibiotics bearing pyrrolidine moiety) lack the inhibition ability against *A. fumigatus,* so they are only used for salvage therapy of invasive aspergillosis. Likewise, the most recommended drugs for both treatment and prophylaxis of aspergillosis include triazole antifungals: itraconazole, voriconazole, and posaconazole (Figure 1)^81^ and again, unfortunately, *A. fumigatus* isolates are becoming globally more resistant to them to a variable extent^82^. The great importance of the triazoles containing drugs against *A. fumigatus* comes from the fact that they are the only drug that can be given orally for long term therapy. The increasing resistance documented of course compromises such clinical progress^15^. Our results also come in agreement with Dabur *et.al*.^83^ that discovered naturally occurring antifungal pyrrole derivatives from Datura leaves that was effective against pathogenic *A. fumigatus*^83^.

It can be concluded from our results against the tested filamentous fungi that our newly synthesised compounds possess a very good and broad-spectrum antifungal activity against both the yeast and filamentous fungi, such a characteristic makes the compounds own an important feature of the ideal antifungal agent according to Mazu *et.al.*^9^.

#### 3.2.5. The effect of the tested compounds on some selected species nucleic acid

To analyse the effect of the tested compounds on the pathogenic filamentous fungal species nucleic acid, we set up a gel electrophoresis analysis.

##### 3.2.5.1 The effect on bacterial nucleic acid

**Figure 3.**
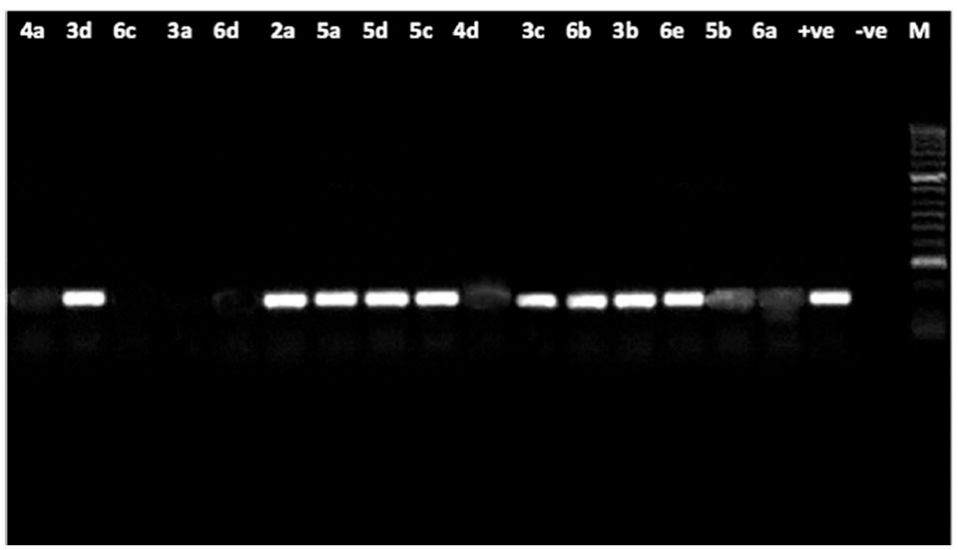
Agarose gel electrophoresis showing the effect of the tested compounds on the nucleic acid of *P. aeruginosa*. From right to left: lane 1marker (M), lane 2 (negative control), lane 3 (positive control) lane 4 to 19 (fungal DNA treated with the synthetic compounds.

*P. aeruginosa* was selected as a representative of the bacteria for this experiment for the following reasons: The Gram-negative bacteria are more resistant than Gram-positive ones, out of the former class comes *P. aeruginosa* as a major cause of the most challenging issue regarding nosocomial infections due to resistance to antibiotics^76^. *P. aeruginosa* resistance is multifactorial and remarkable against many of the currently used antibiotics making them ineffective. The effect on the DNA of *P. aeruginosa* ATCC 27853 is shown in Figure 3.

The results showed intact DNA bands with the untreated DNA (positive control), where no significant damage occurred. The same was shown with nine of tested compounds, treated DNA showed intact bands like the untreated DNA, which means that those compounds have no effect on DNA, maybe because of a developed mechanism of resistance, and also they could be acting through any other mechanism. In contrast, compounds **3a, 4a, 4d, 5 b** and **6a,c, d** showed substantial alteration in electrophoretic migration with compounds **3a, 6c** showing the highest effect, which suggests that the antimicrobial effect of them occurs by a direct chemical damage to DNA.

***To Discuss the former results*;** Comparing the MIC values of the compounds against *P. aeruginosa* (Tables 1 and 2) with their results on the bacterial DNA (Figure 3) showed that, first: some compounds (namely; **3a, 4a, 4d, 5 b** and **6a,c, d**) could exert their antibacterial effect on *P. aeruginosa* through damaging its DNA, and second: as the compounds have closely related MIC values, yet very different effect on its DNA, this leads to the conclusion that the effect on the bacterial DNA is mostly not the main mode of antibacterial action of all the examined compounds and there must be other targets inside the *P. aeruginosa* bacterial cell that get affected by those compounds. It can also be concluded that *P. aeruginosa* developed a resistance mechanism such as; an efflux pump, modification of target or deactivation of the chemical compound, that protected its DNA from compounds revealed no effect on it.

The effect shown by the tested compounds on the DNA of *P. aeruginosa* comes in agreement with the well-known effect of many antibiotics on the bacterial DNA and the possible mechanism could be related to any of the several mechanisms exhibited by them. For example, there is a recently reported mechanism of DNA acting antimicrobials active on Gram-positive bacteria through inhibiting the DNA polymerase IIIC^84^, and another possible mechanism could be through affecting the DNA primase^85^. Usually, the bacterial DNA damage caused by antibiotics can be exerted either by direct chemical modification inducing breakage in DNA, like metronidazole (imidazole derivative) or through affecting the enzymes associated with its replication. To the second class belong: quinolones, coumermycins and novobiocin. The quinolones selectively inhibit DNA gyrase (topoisomerase II) leading to chromosomal fragmentation. Quinolones include nalidixic acid and the newer second-generation fluoroquinolones (FQ): norfloxacin and ciprofloxacin. FQ are much more effective^86^ and are able to inhibit all Gram-negative organisms, including Pseudomonas species^87^ with ciprofloxacin coming as the current first choice against them^86^. The coumermycins and novobiocin also inhibit DNA gyrase but with a mechanism different from quinolones^88^^,^^89^. FQs are among the most widely used antimicrobials on the market^90^, yet the increasing resistance to it urged the development of novel topoisomerase inhibitors^91^.

A question may arise about the effect that the compounds may have on the human DNA, but since the protein importance in the bacterial DNA replication is different from the eukaryotic counterparts, chromosome replication represents a promising therapeutic target^90^^,^^91^ without such a fear on human safety^92^. There are many examples about the selectivity of the DNA acting antibacterial agents based on the structure difference of the targeted enzymes between bacteria and human, like quinolones which do not interfere with the human topoisomerase enzymes^93^, bacterial primase inhibitors^85^ and Pol IIIC inhibitors^84^.

##### 3.2.5.2. The effect on the filamentous fungal nucleic acid

**Figure 4.**
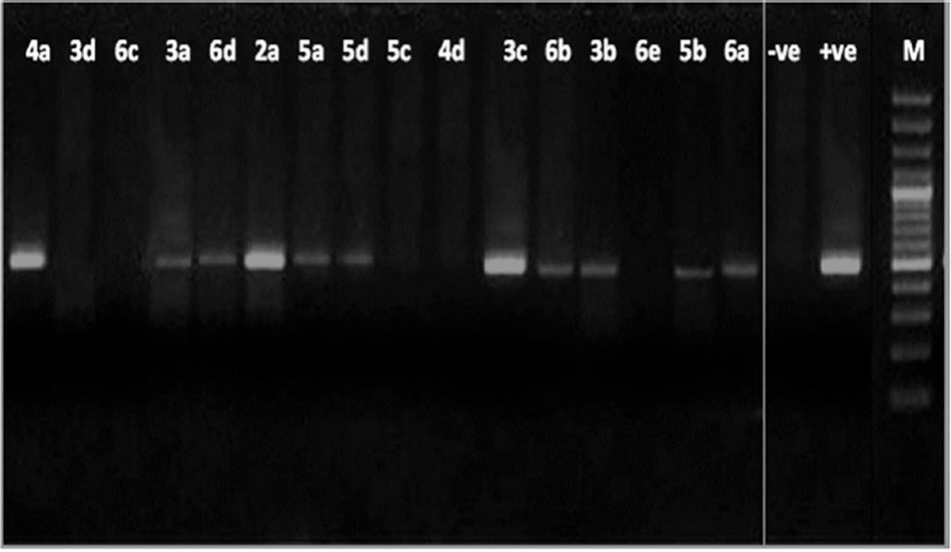
Agarose gel electrophoresis showing the effect of the tested compounds on the nucleic acid of the pathogenic fungus *F. oxysporum*. From right to left: lane 1marker (M), lane 2 (positive control), lane 3 (negative control) lane 4 to 19 (fungal DNA treated with the synthetic compounds.

The effect on the DNA of the pathogenic fungus *F. oxysporum* is shown in **(**Figure 4**).**

The results showed intact DNA bands with the untreated DNA (positive control), where no significant damage occurred. DNA treated with compounds **2a, 3c and 4a** also showed intact bands like the untreated DNA, which means that those compounds have no effect on DNA and must be acting through any other mechanism. In contrast, the remaining tested compounds showed substantial alteration in electrophoretic migration with compounds **3d, 4d, 5c, 6c and 6e** showing the highest effect.

***The former results indicated that*;** most of the compounds (thirteen out of sixteen examined compounds) could be exerting their antifungal effect by direct chemical damage to the DNA or at least it is one of the target sites. Comparing the MIC values of the compounds against *F. oxysporum* (Table 3) with their results on the fungal DNA **(**Figure 4**)** shows that two compounds (**2a** and **3c**), which showed the (lowest and highest) antifungal activity, respectively, are showing the same effect on DNA which is no damage, such an observation can suggest that the effect on DNA is not the MAIN mode of action of at least these two compounds or that there may be a strong resistance mechanism inside the fungal cell that leads to decreasing their effect on DNA like efflux pump, change in the target site, deactivation of the compound, or decreased permeability ^76^.

Comparing Figures 3 and 4 showed that the effect of the examined compounds on the DNA of the filamentous fungi is of much wider range than that on *P. aeruginosa* (13 compounds are effective **(**Figure 4**)** in comparison to only seven **(**Figure 3**)**. Also, from the same comparison, it can be shown that there is an agreement between the MIC values and the effect on the DNA in case of *F. oxysporum* of more compounds **(**Figure 4**)** than what’s seen in case of *P. aeruginosa* **(**Figure 3**)** which means that still the effect on DNA is more common in fungi and it could have a bigger role in the antifungal effect than the antibacterial one of the tested compounds.

If the antimicrobial drug is affecting the DNA of the filamentous fungi, which are eukaryotes, must it affect the human one too? actually the answer is: not necessarily, because the literatures show that there are important differences between mammalian and fungal cell types and such differences have already enabled researchers to develop some drugs like 5-fluorocytosine (5FC), that interferes with pyrimidine metabolism, RNA, DNA, and protein synthesis of fungi, yet it’s safe for human use due to the enzyme differences. Topoisomerases I (TOP) inhibitors represent another successful example and the reason is that it affects the enzymes of *C. albicans* more than the human ones like aminocatechol A-3253^5^.

Several antifungals target fungal DNA or RNA like a natural product called Yatakemycin that acts by sequence-specific DNA alkylation. Icofungipen is another example, it is an orally administered, synthetic derivative of the naturally occurring β-amino acid cispentacin, blocks isoleucyl-tRNA synthetase, which leads to the inhibition of protein synthesis and growth of fungal cells^94^. Other products that give their antifungal activity by interfering with the fungal cell nucleic acid biosynthesis are some natural products; sampangine, meridine, onychine and berberine^5^. Of course in order to be completely sure that our compounds are safe for human use further investigations on their toxicity profile will be needed in future work.

Some possible effects of pyrrole could also be on the synthesis of proteins; for example: the antibacterial properties of some halogenated pyrroles were due to their ability to affect the synthesis of macromolecules in Gram-positive and Gram-negative bacteria^95^. Also, A pyrrole derivative exhibited its antifungal activity against *A. fumigatus* by inhibiting the expression of two important proteins at the time of spore germination^83^. Other pyrroles affected proteins but not at the level of synthesis as protein inhibitors like toyocamycin and sangivamycin, two pyrrole derivatives that are reported as inhibitors of protein kinase C and/or protein kinase A^50^ and also several other pyrrole derivatives were found to be inhibitors of several types of protein kinases^27^. There are other antifungal agents reported to produce their effect through acting on some proteins, Sordarins are one example that acts on the yeast protein synthesis elongation cycle. Elongation Factor-3 is required by the 40S ribosomal subunit of yeast but not by human cells so the inhibition happens with no effect on the protein synthesis in human. Also some antibiotics with antifungal activity (Enactins and neoenactins) affect the protein post-translational modification by inhibiting the protein-*N*-myristoyltransferase (NMT). NMT has different substrate specificities in fungi and mammalian cells, making it a good target for designing antifungal agents against fungi that requires it like *Cryptococcus neoformans* (*C. neoformans*). Another inhibitors of NMT, the analogs of 2-bromotetradecanoic acid, were active against filamentous fungi such as *A. niger* and also on yeasts like *S. cerevisiae*, *C. albicans* and *C. neoformans*^5^.

We tried to find out a possible mechanism that is responsible for the DNA damage inside the filamentous fungi that was produced by our compounds in this study; an interesting finding was about the reactive oxygen species (ROS). ROS are molecules with unstable highly reactive free radicals, they get produced during normal cell routine or as a response to exposure to drugs then when they reach high levels they can damage the cellular DNA^96^.

Such a finding was assumed to be the possible mechanism because literatures have a lot of examples of antifungals that cause increased ROS in fungal cells, for example,^97^^,^^98^ showed some antifungals that lead to intracellular production of ROS in filamentous fungi such as *A. fumigatus.* Also, miconazole use was reported to generate ROS in *C. albicans*^14^^,^^98^, Finally, there is fluconazole (FLC) that leads to generation of ROS in *C*. *albicans* neoformans^96^.

Pyrroles bearing an imidazoline group at C-3,were prepared and examined as an investigation for new anti-microbial agents, especially as an anti-candida^99^. The anti-microbial activities of these compounds indicates; higher anti-candida activity over the **Amphotericin B,** one of the most active antifungal agent against most Candida species^6^. Compounds **2a, 3c** and **4d** have a very good activity against both Gram-positive bacteria and candida but that the anti-candida effect is higher than the antibacterial one, owning these activities to the pyrrole moiety.

**Figure 5.**
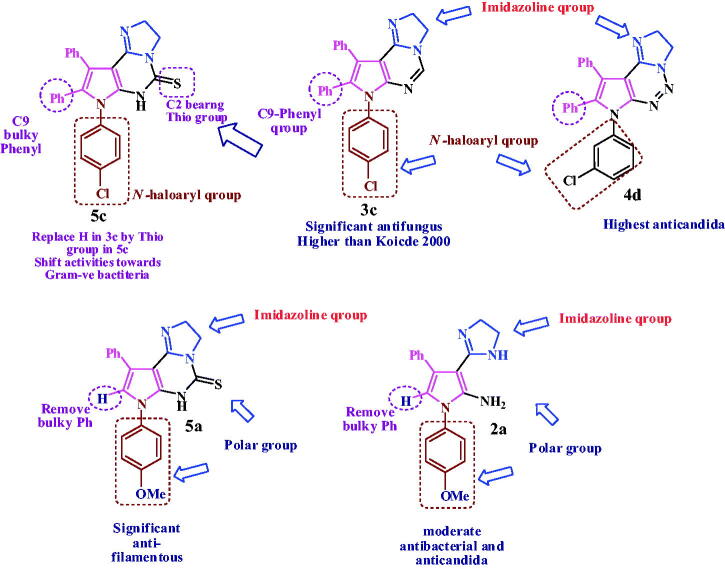
Relation between chemical structure and microbial activity of compounds (2a, 3c, 4d, 5a and 5c).

The highest active compound for both Gram-positive and filamentous fungi as plant pathogen is; pyrrolopyrimidine **3c** bearing imidazoline, phenyl at C9 and *N*-haloaryl. Replace the pyrimidine with triazine ring as in **4d** shift the activities against fungi, with enhancing the antibacterial and anti-candida activities. Replace the C9-bulky phenyl in **3c** with H and *N*-haloaryl with hydrophilic *N*-methoxyphenyl group mask the bacterial and yeast activity, but enhance the antifungal activity as in thio derivative **5a (**highest activity against filamentous as human pathogen**).** Another thio derivative, bearing *N-*haloaryl and phenyl at C9, compound **5c** shown the highest activity against Gram-negative bacteria. Three fused ring exhibit a predominant activities over the free pyrrole bearing imidazoline at C2 as in compound **2a** which show moderate activity against Gram positive and yeast, as shown in Figure 5.

## Conclusion

In this study, we have presented novel 3-imidazolyl pyrroles, and their fused form (pyrimidines and triazines) that can act as both antibacterial and antifungal agents. Compounds **2a, 3c, 4d and 5c** showed a very good activity against Gram-positive bacteria in comparison to ciprofloxacin with higher MZI and lower MIC values. Compound **5c** was the best against Gram-negative bacteria with MZI value comparable to that of ciprofloxacin, an equal MIC against *P. aeruginosa* ATCC 27853 and an even lower one against *E. coli O157:H7* ATCC 51659. Against *C. albicans,* the effect of compounds **2a, 3c and 4d** exceeded one of the most active antifungal agents, **amphotericin B,** giving higher MZI values; moreover, their MIC values showed several fold decrease than **amphotericin B** up to fivefold. The effect of **3 b, 3c, 5a, 5 b, 6 b and 6e** against the filamentous fungal human pathogens was highly equivalent to that of itraconazole at even a lower concentration (1 µg/mL) with compound **5a** showing the highest inhibition rate **%** (88.21 ± 0.4). The outcomes of this study present some potential candidates that might participate to find a solution to the continuous huge problem of antibiotic resistance both bacterial and fungal and the high need to develop new treatment options for opportunistic infections. In addition, a lot of our synthetic compounds showed potential protection of plants from filamentous fungal pathogens, with a much less toxicity profile that may have a very good agricultural economic impact as they exhibited the desired antifungal activity at a much lower concentration (50 µg/mL) than **Kocide 2000 (2500 **µg/mL), and compound **3c** was the best candidate with the highest inhibition rate (**%**) (**89.9 ± 1.5**). We also propose a possible target of these derivatives inside the bacterial and fungal cells, which is the DNA of the filamentous fungi and *bacteria.* Yet, further investigation about the exact mode of action is required.

## Supplementary Material

Supplemental Material
