# Supplementary figures and images for: Synthesis of novel pyrroles and fused pyrroles as antifungal and antibacterial agents
Source: J Enzyme Inhib Med Chem. 2021 Oct 4;36(1):2183–98. doi: 10.1080/14756366.2021.1984904 (PMC8491725; doi:10.1080/14756366.2021.1984904)

2a

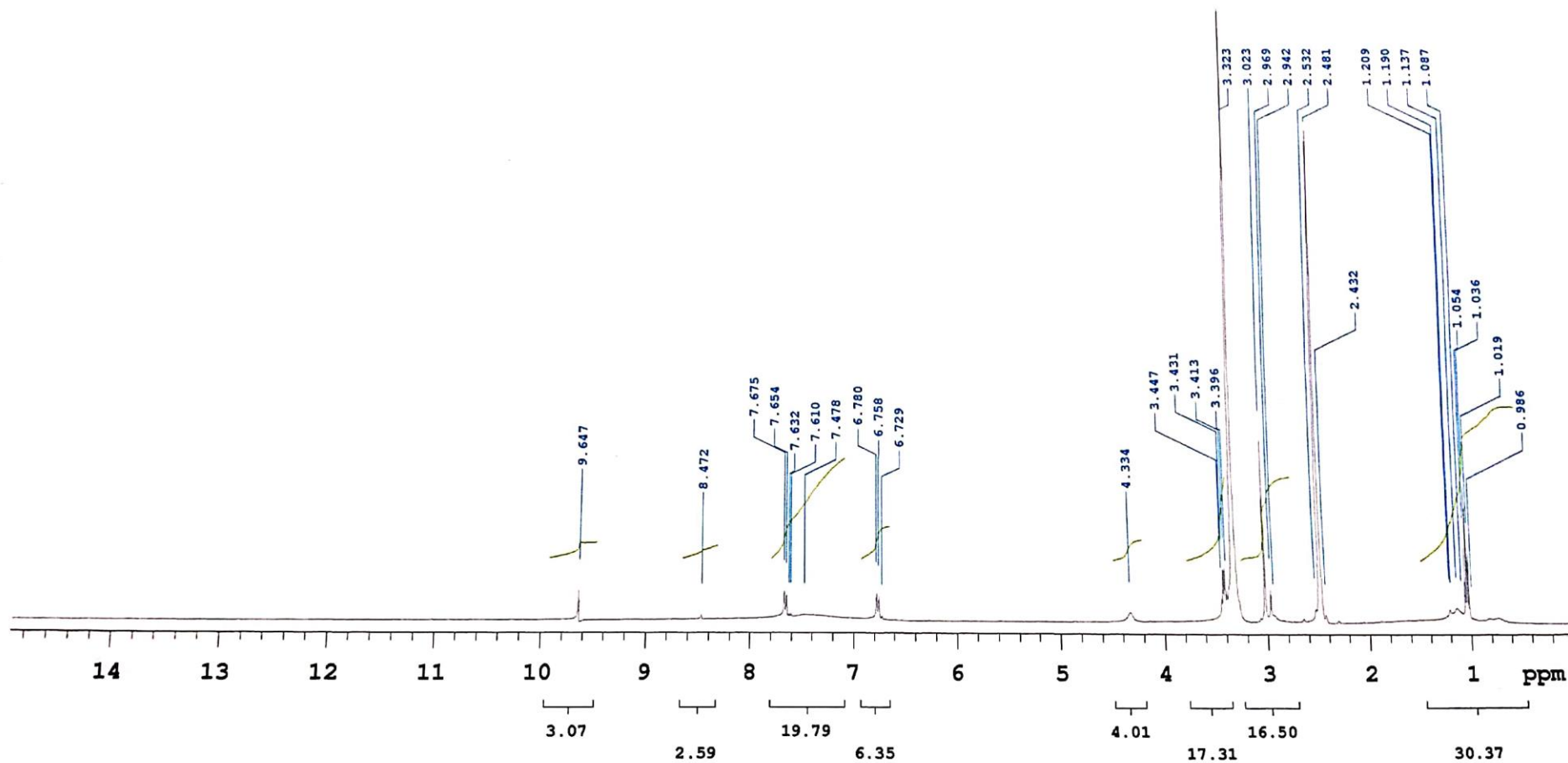

**2b**

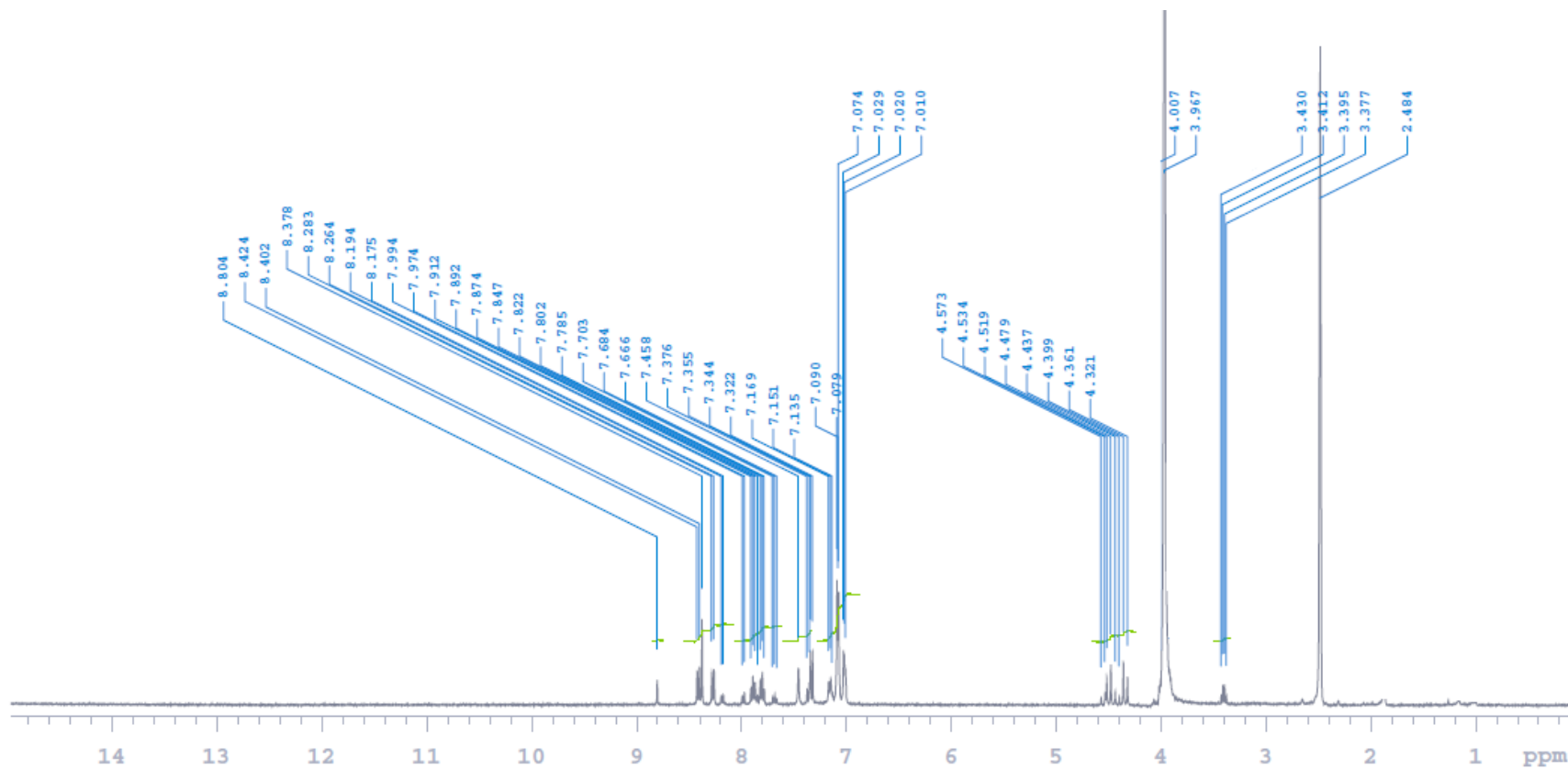

2c

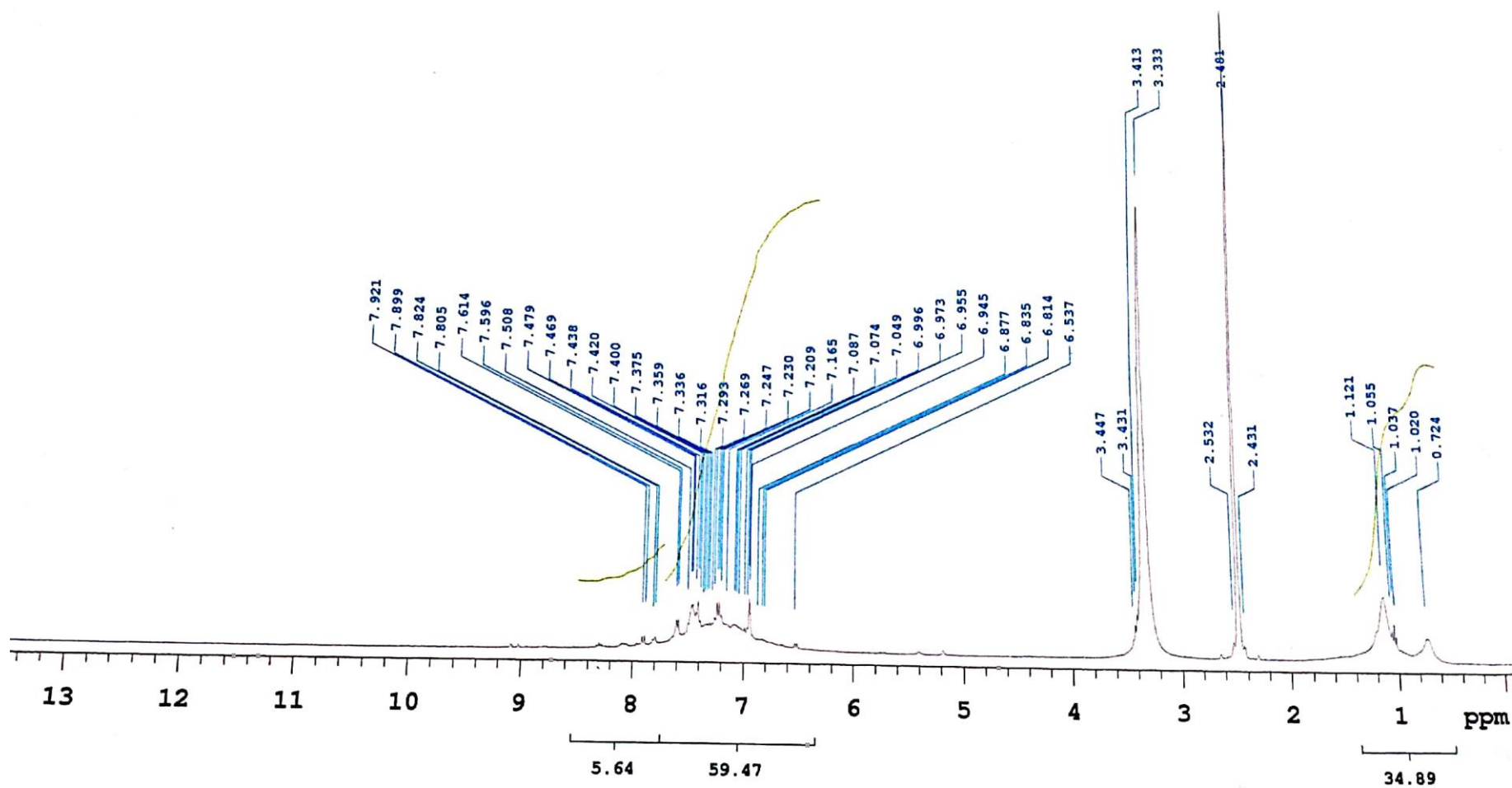

2d

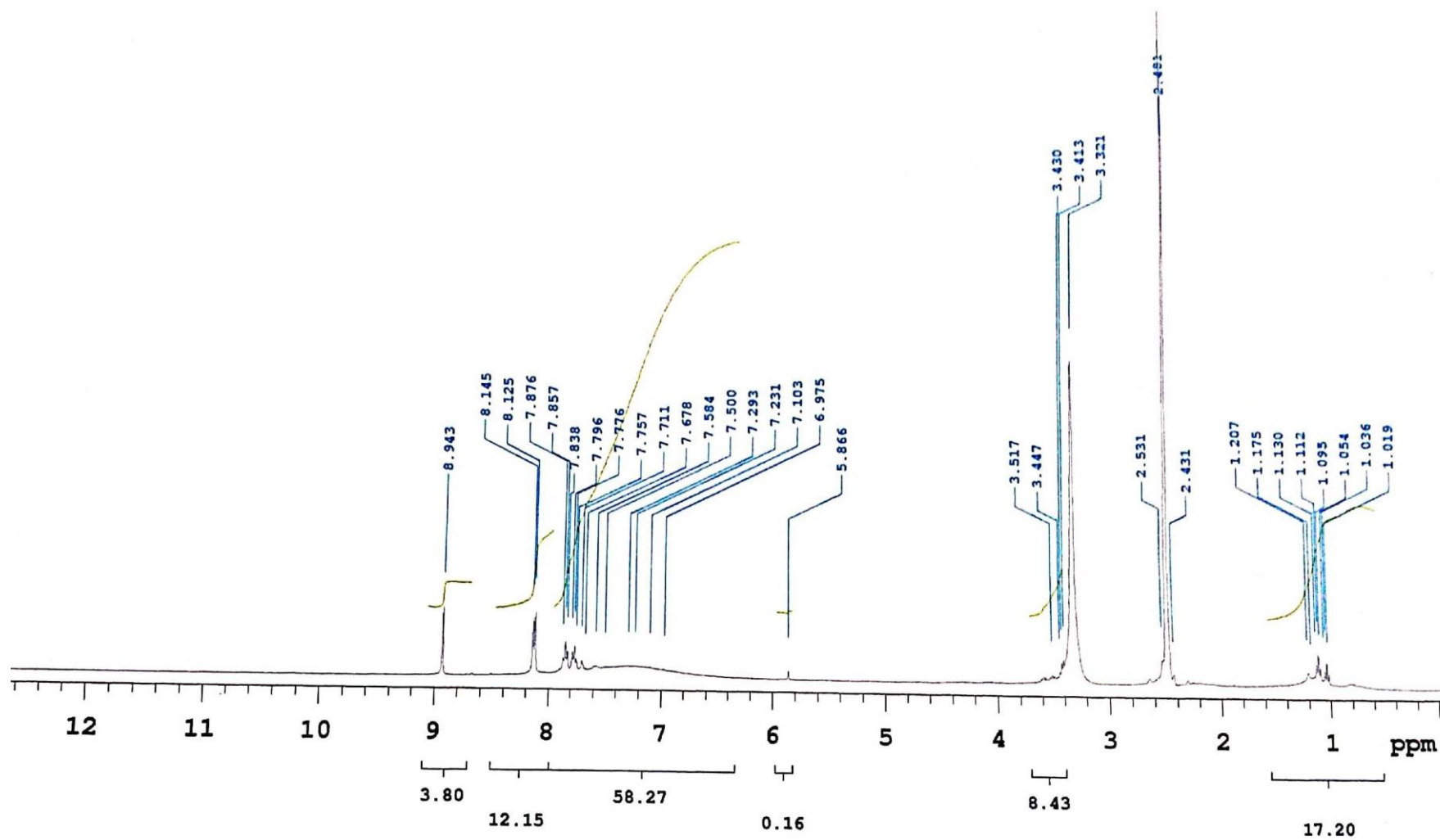

3a

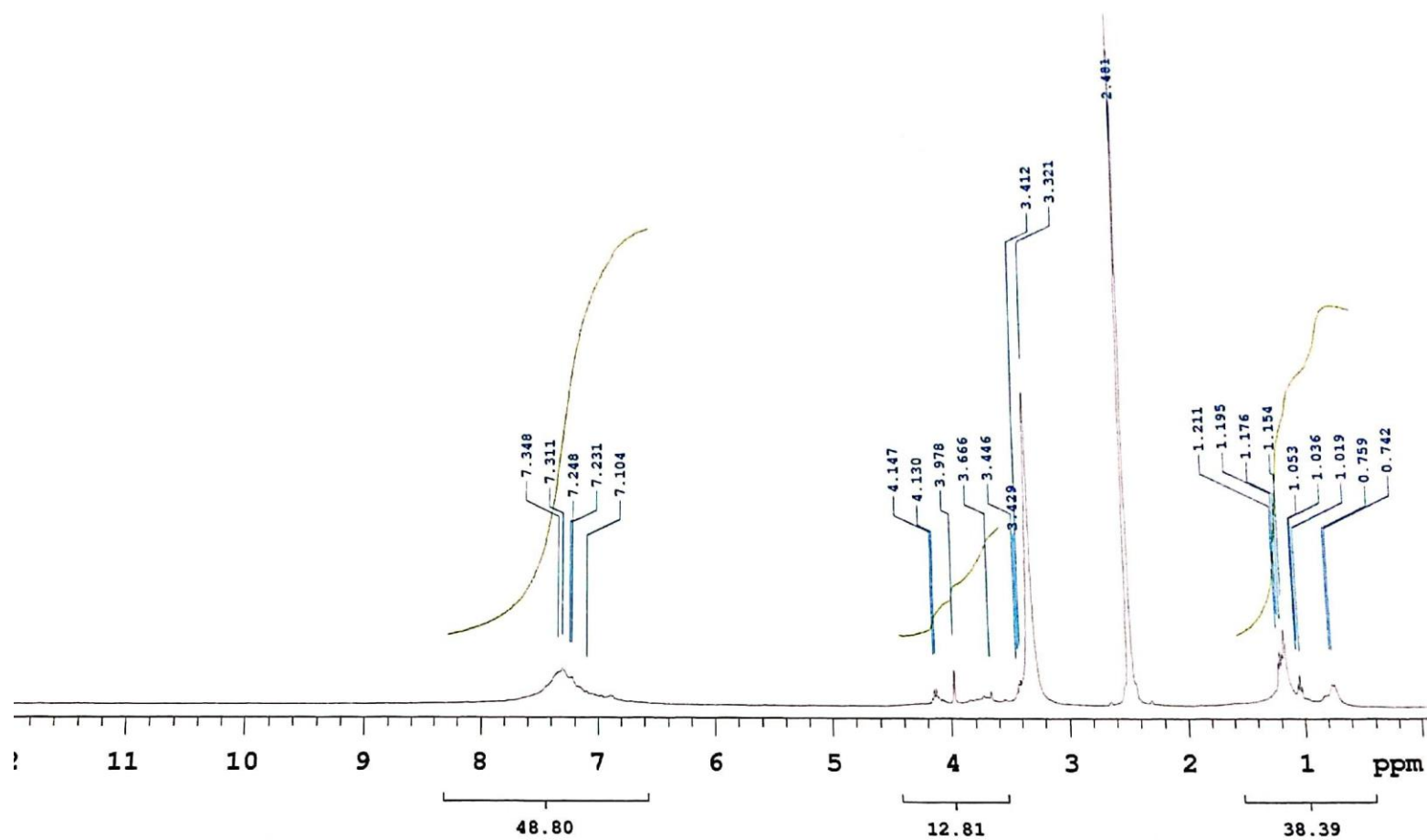

3b

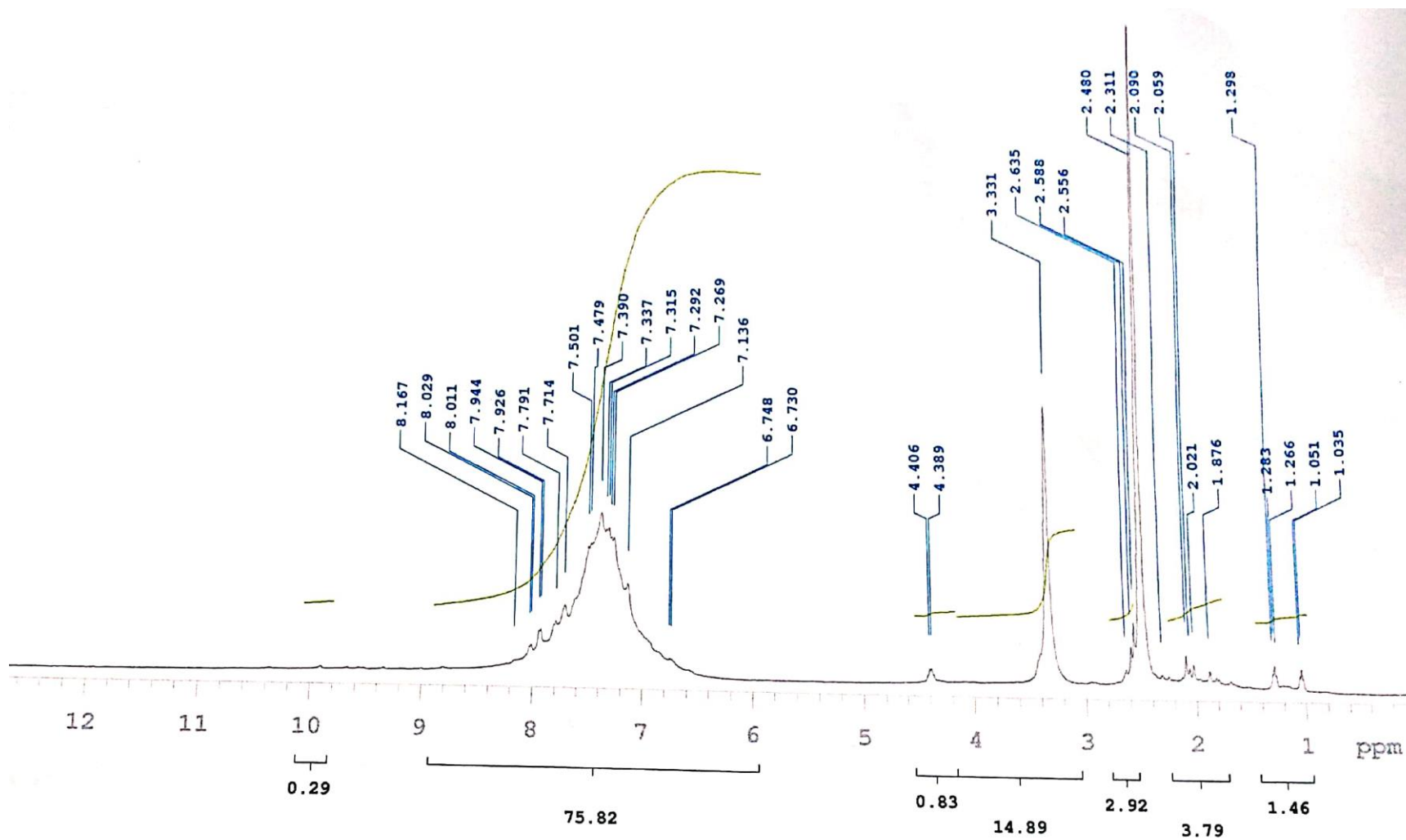

3c

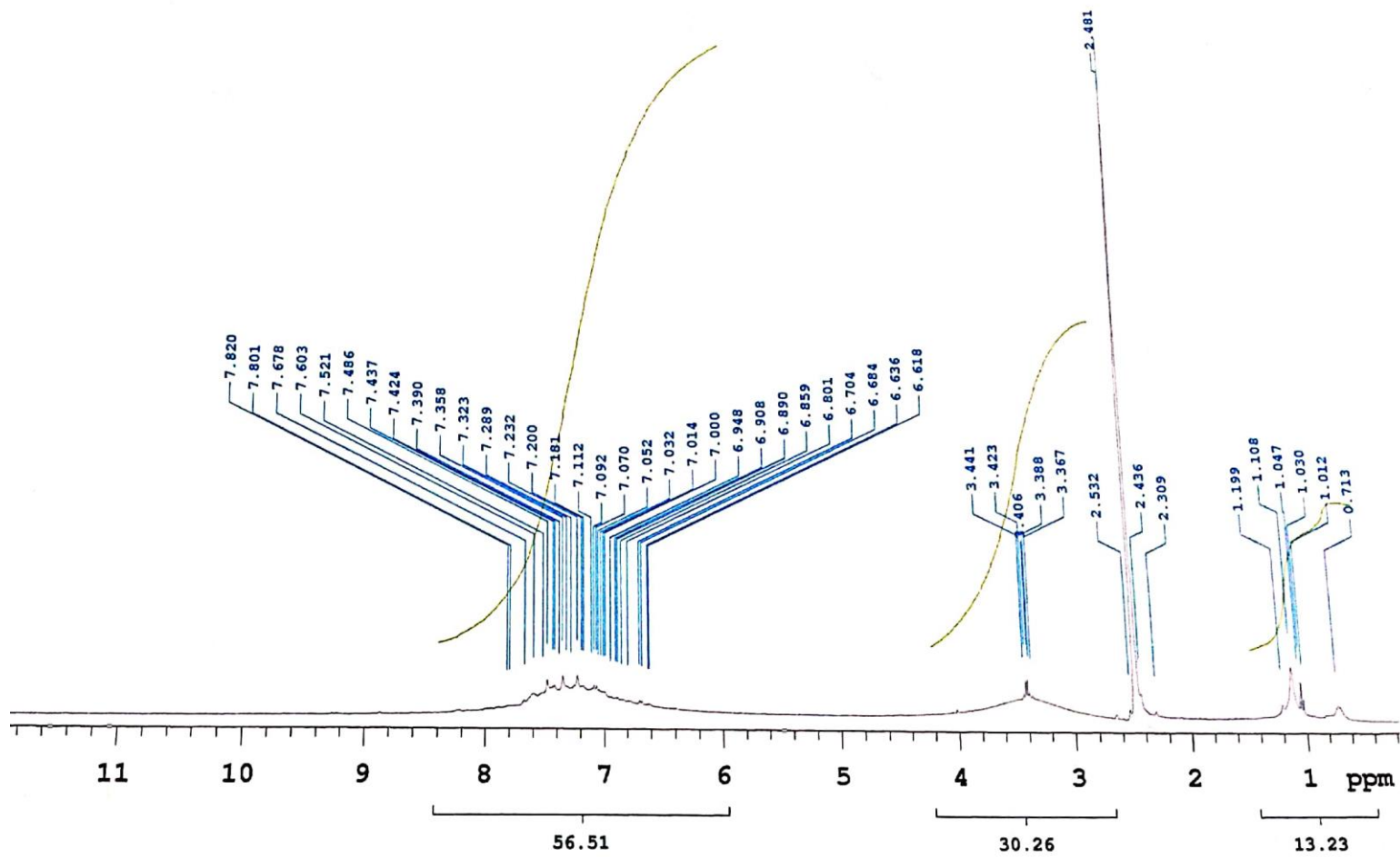

3d

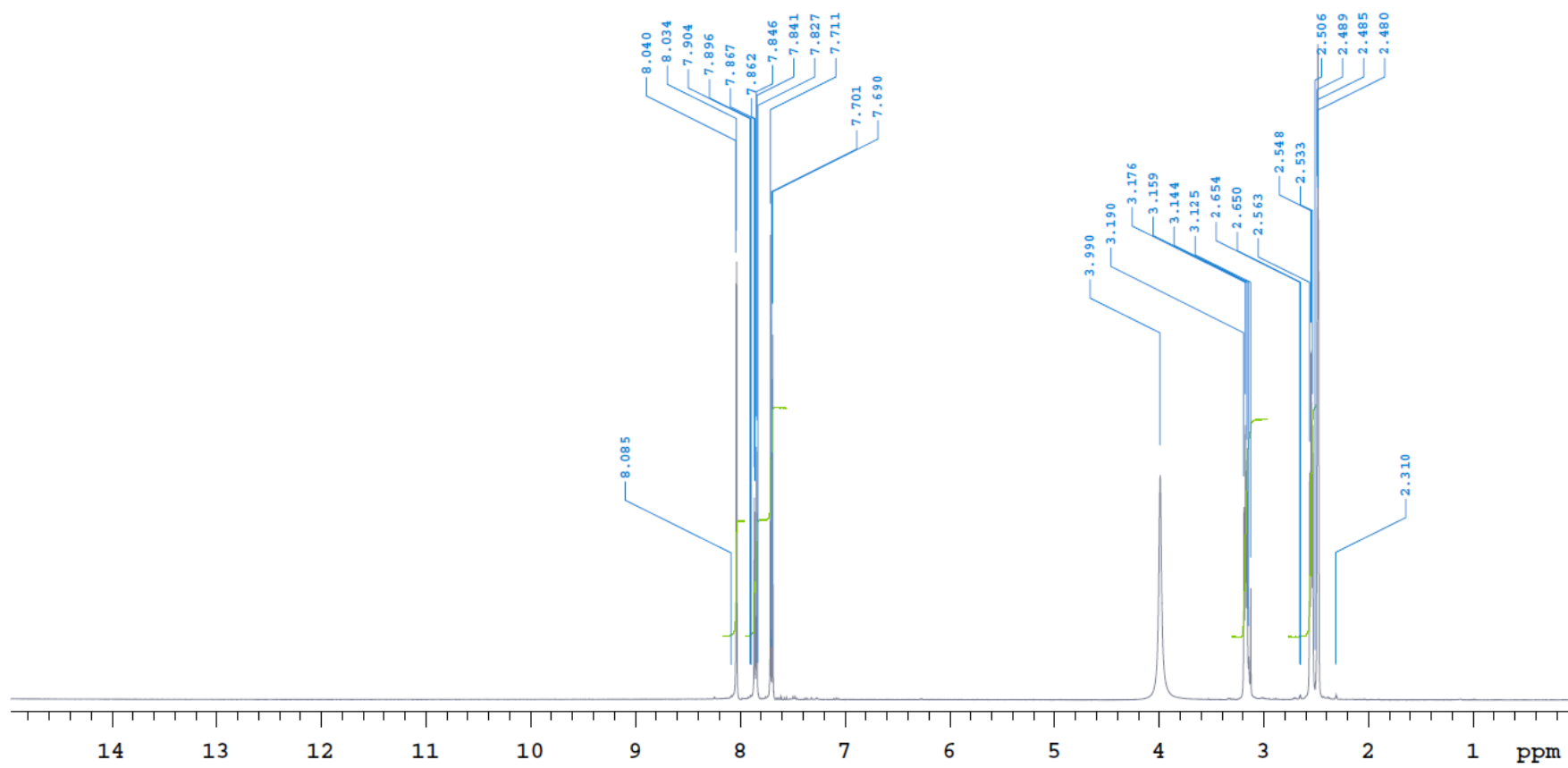

**4a**

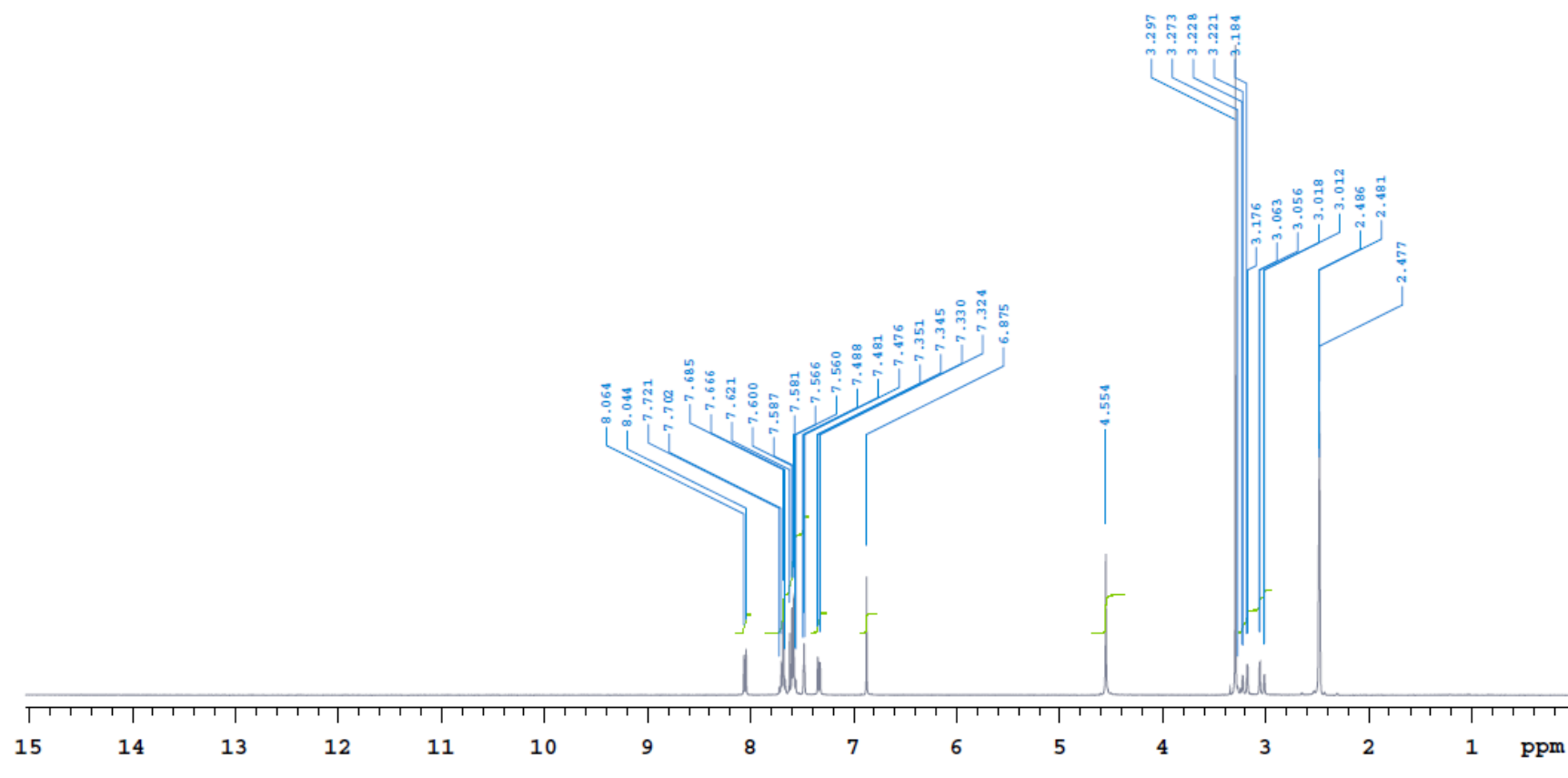

**4b**

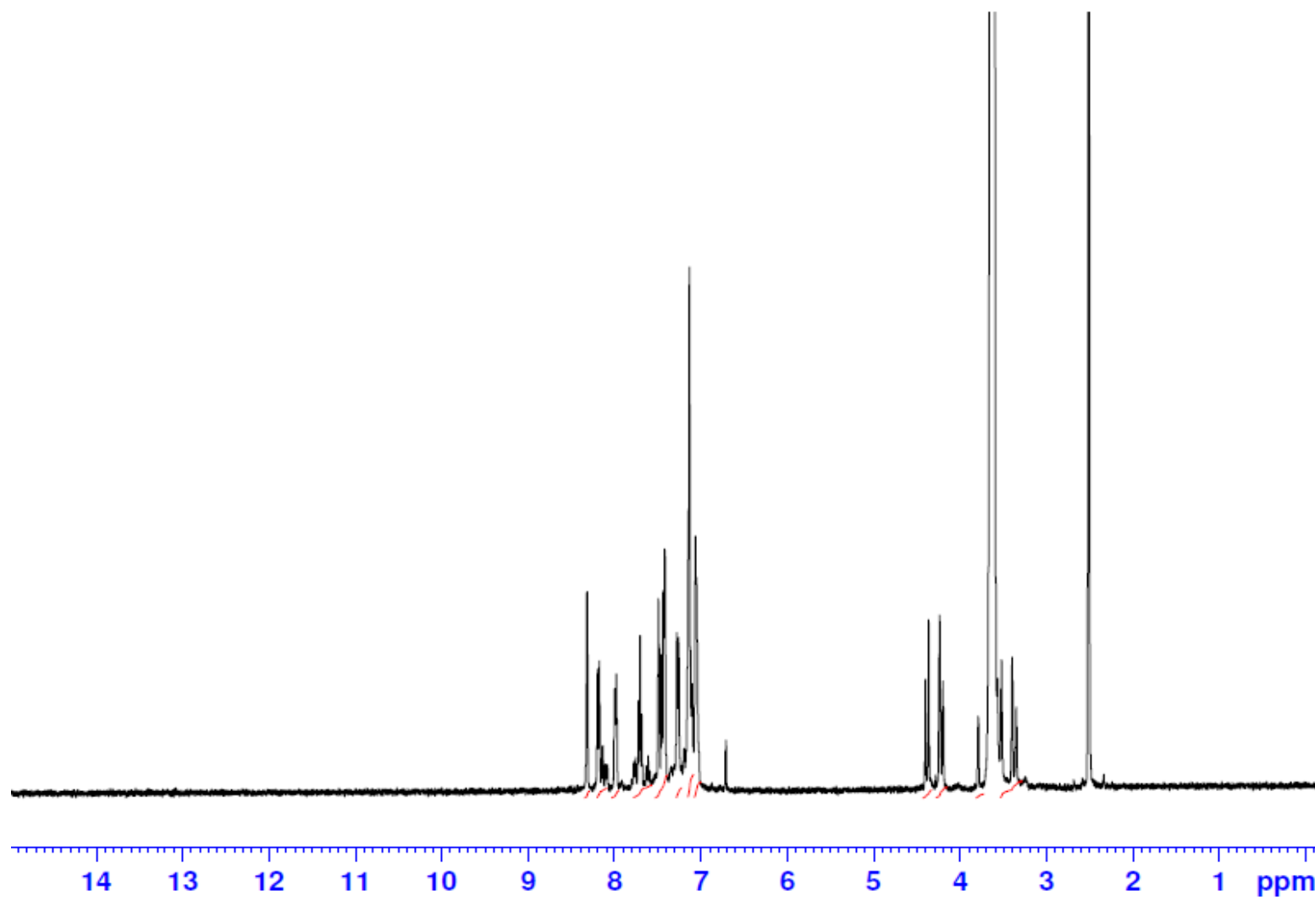

**4c**

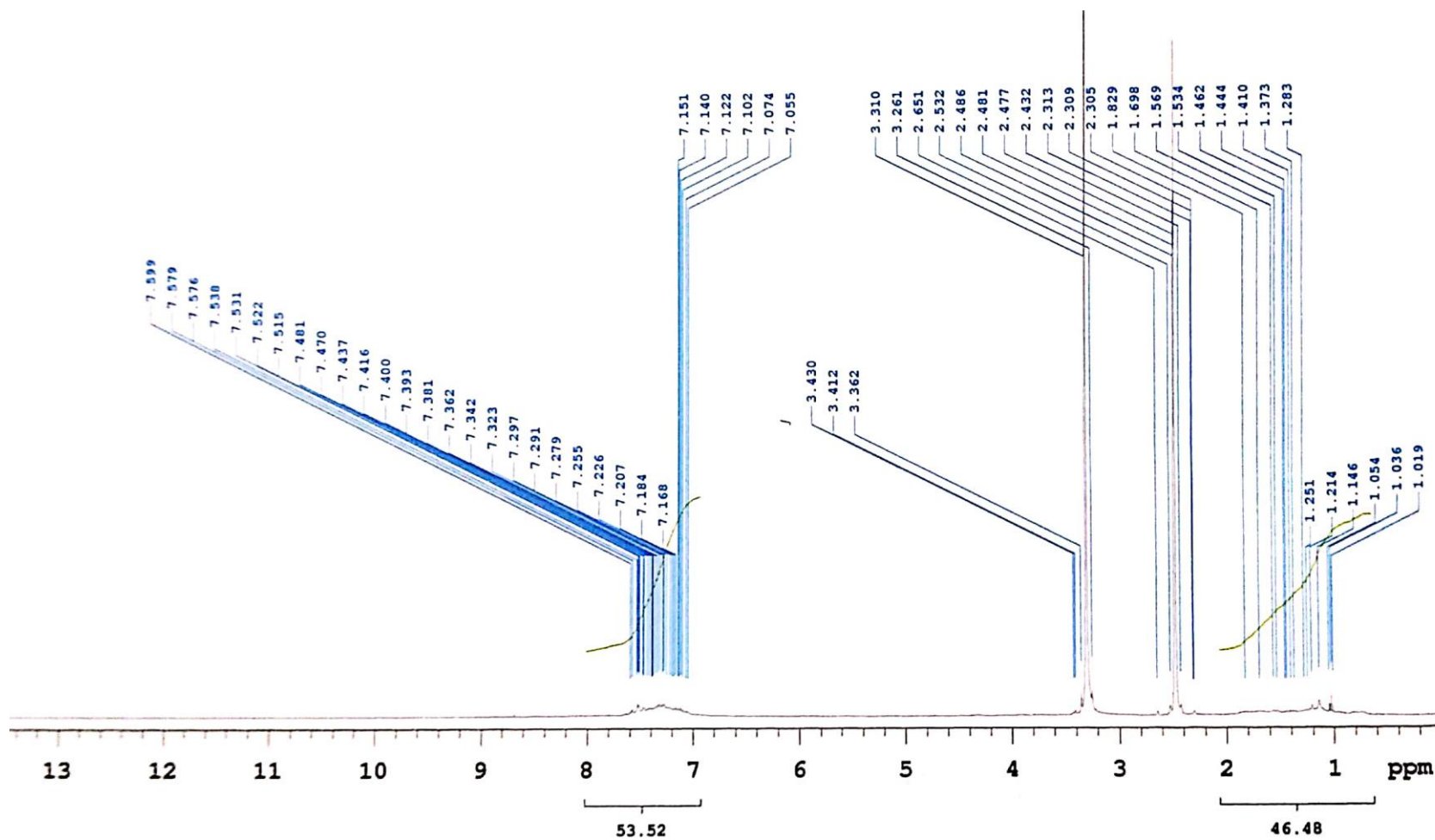

**4d**

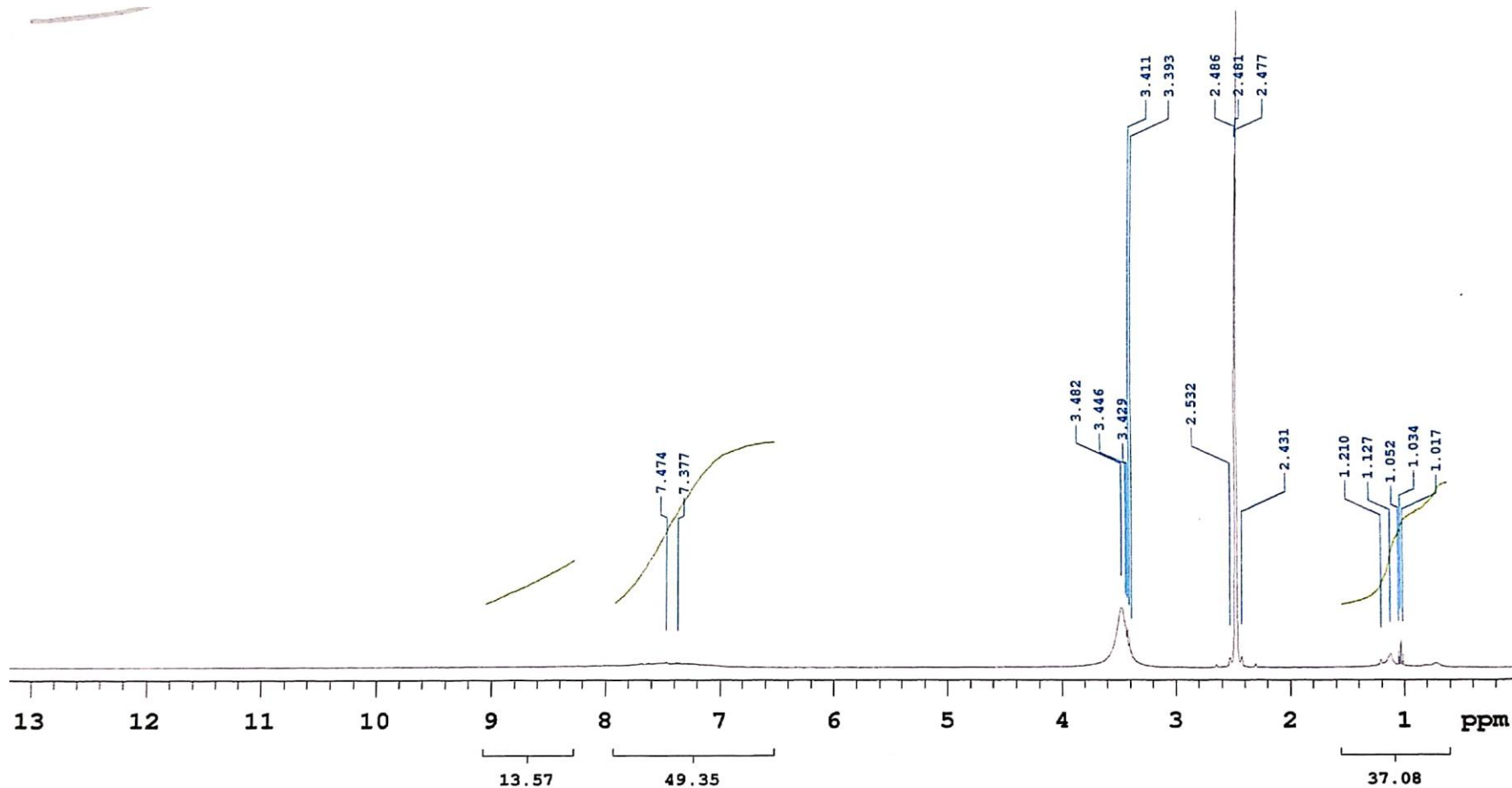

**5a**

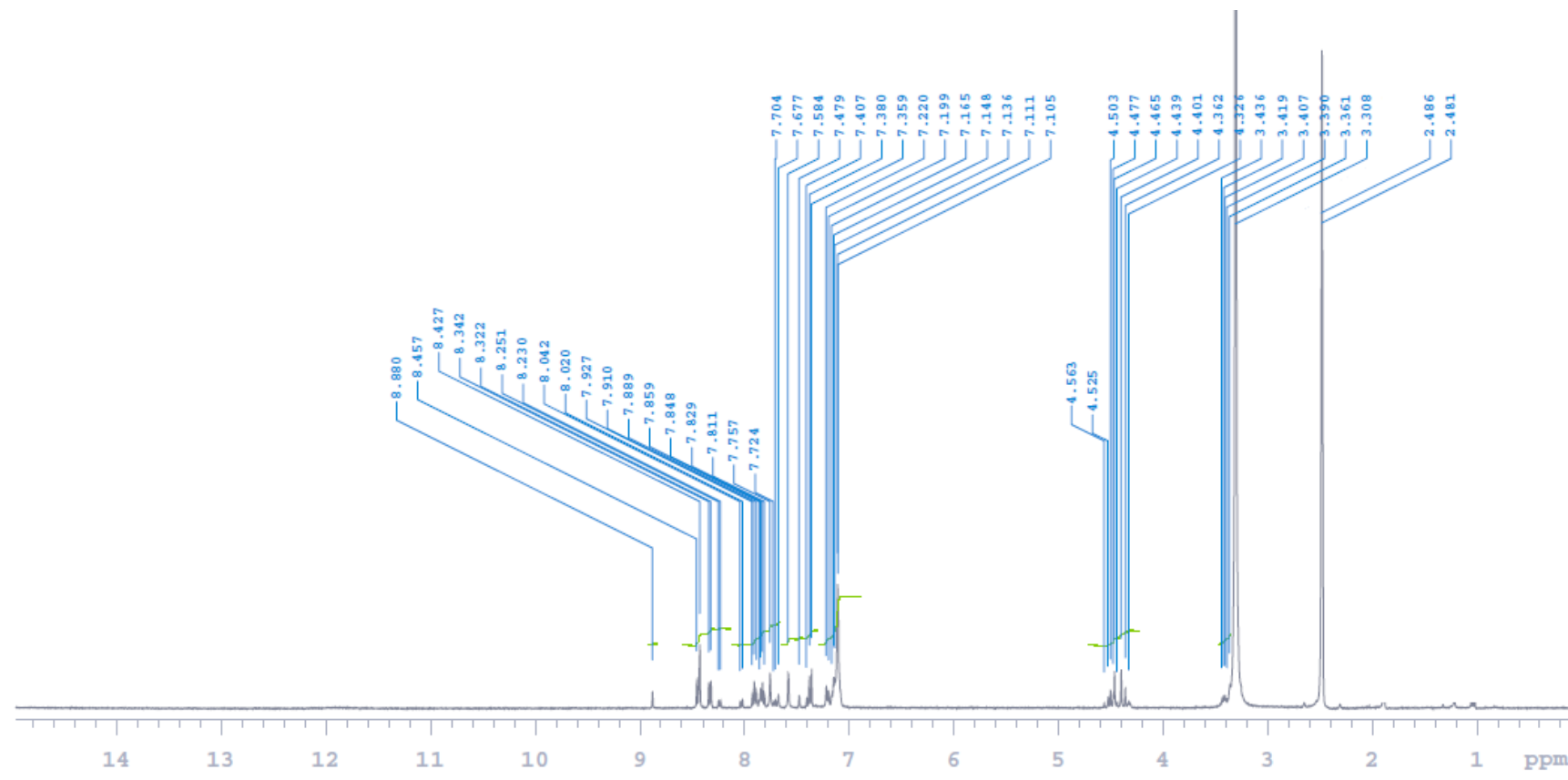

5b

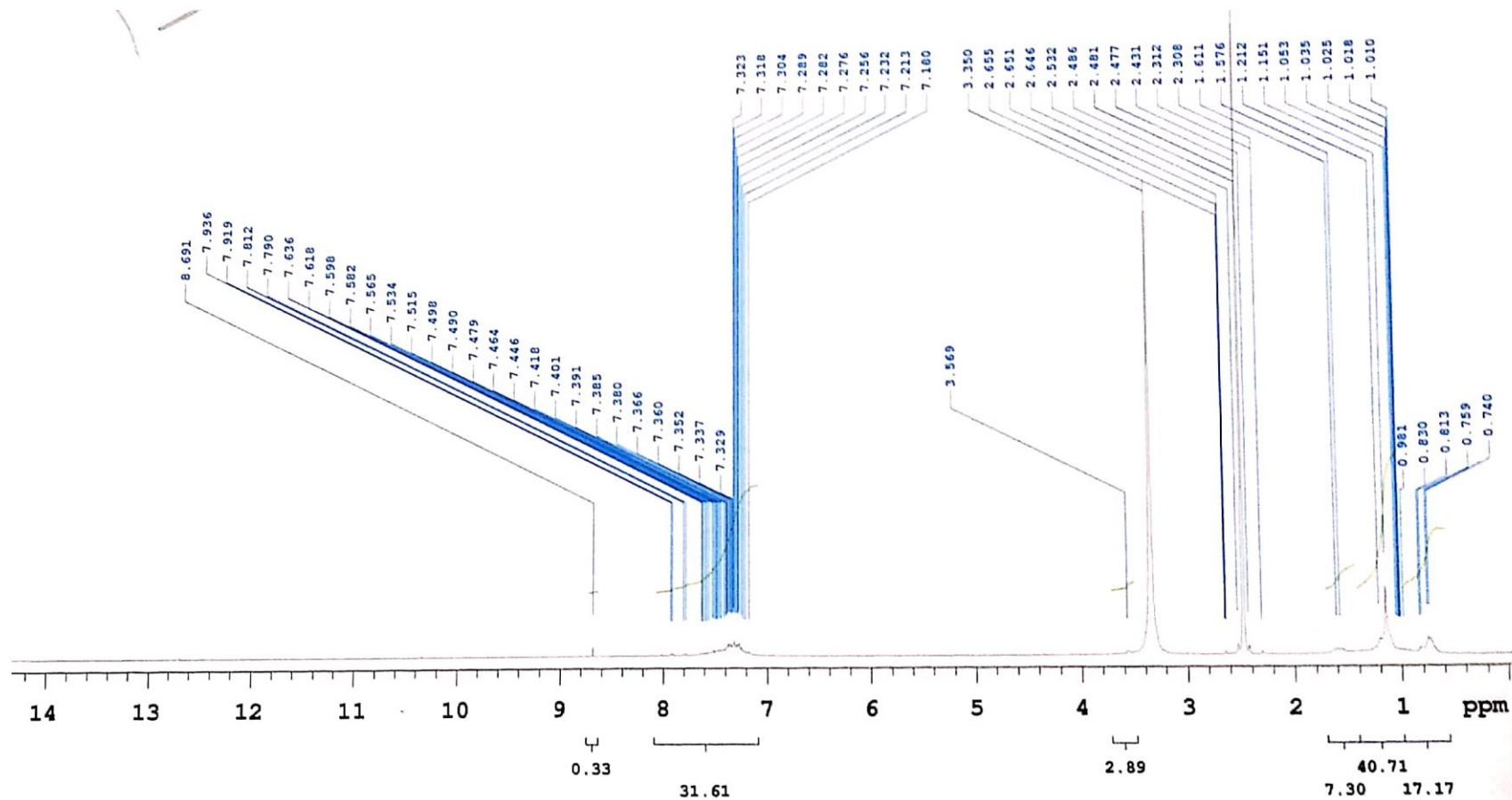

5c

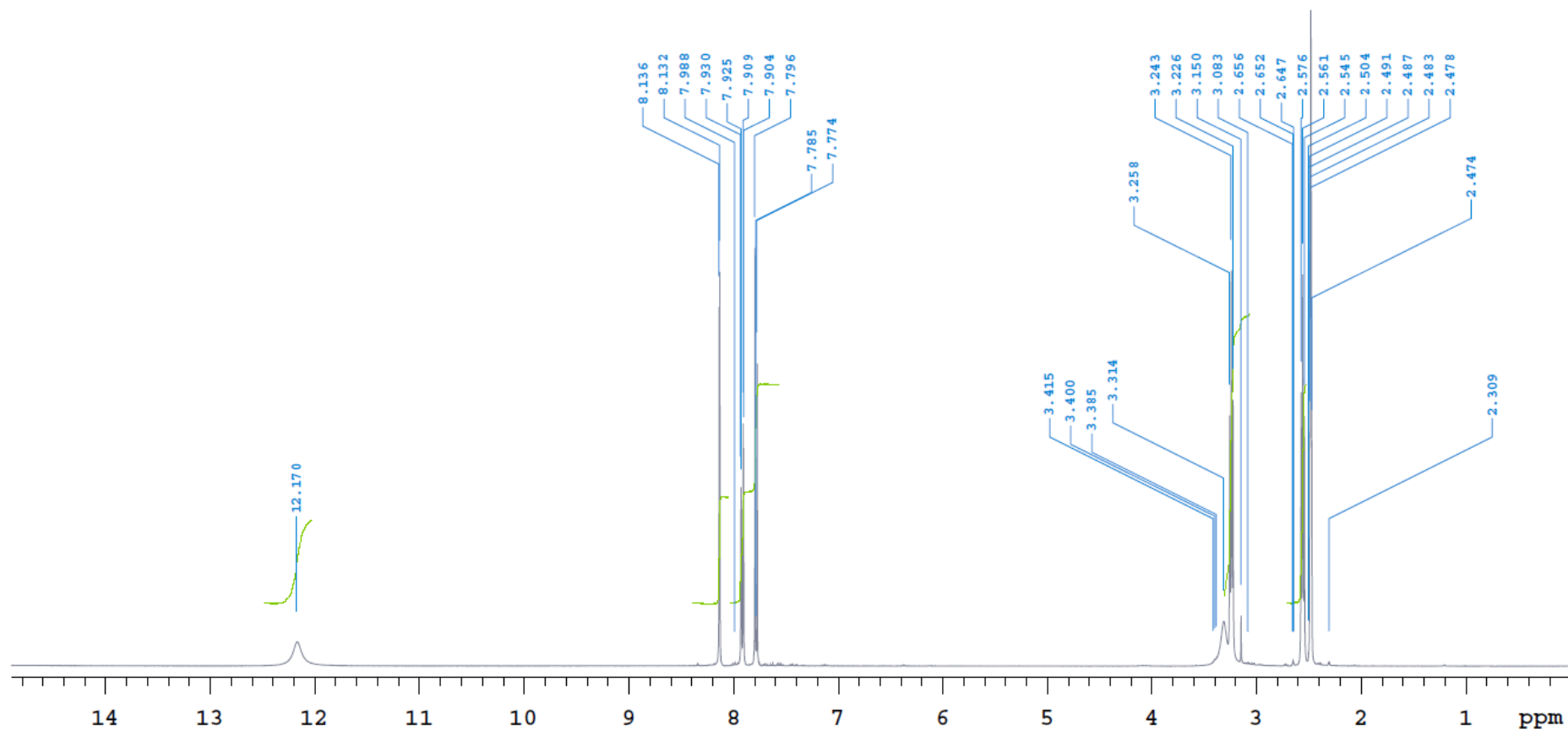

5d

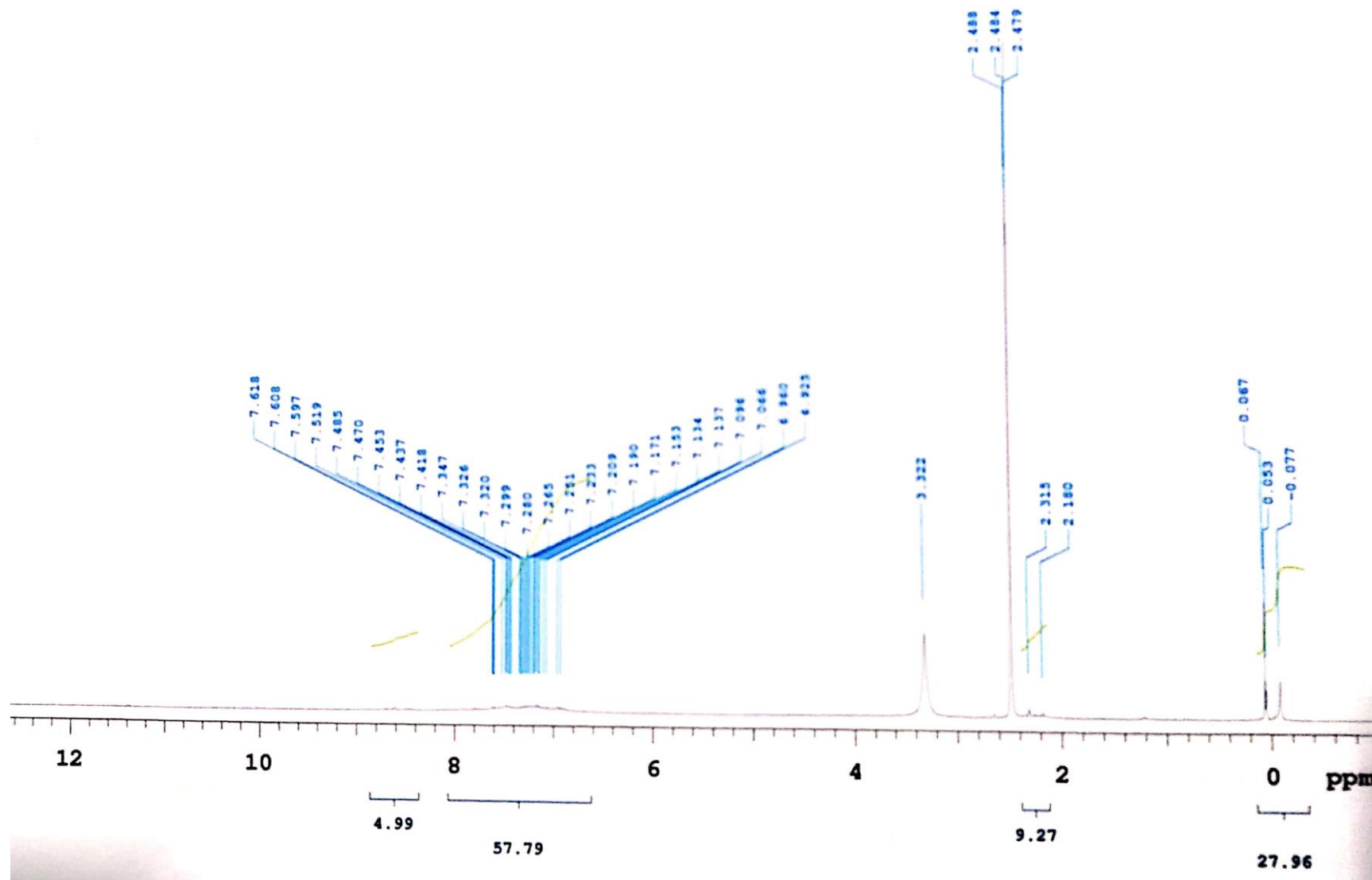

**6a**

iraIbrhime-ED2

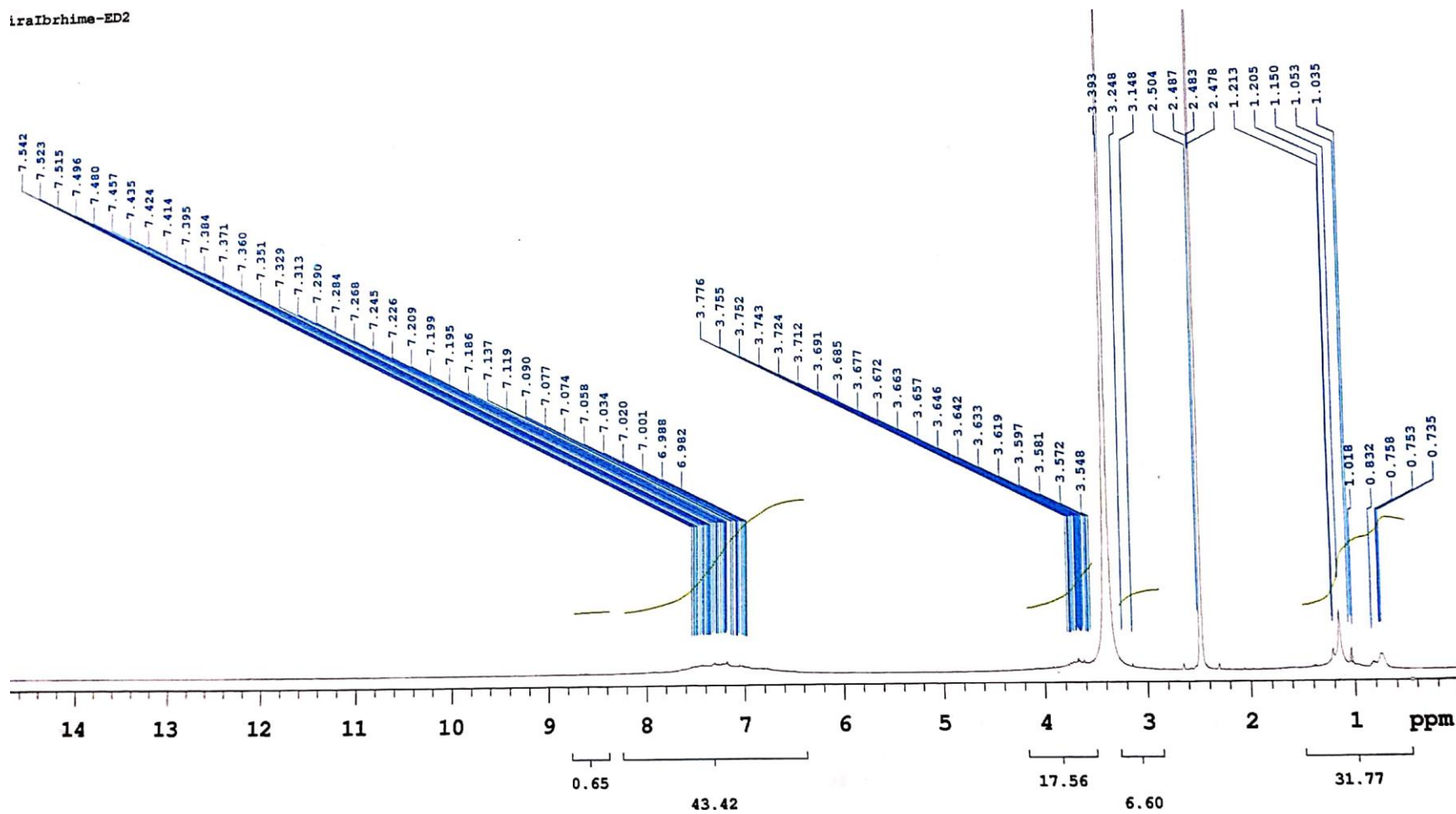

**6b**

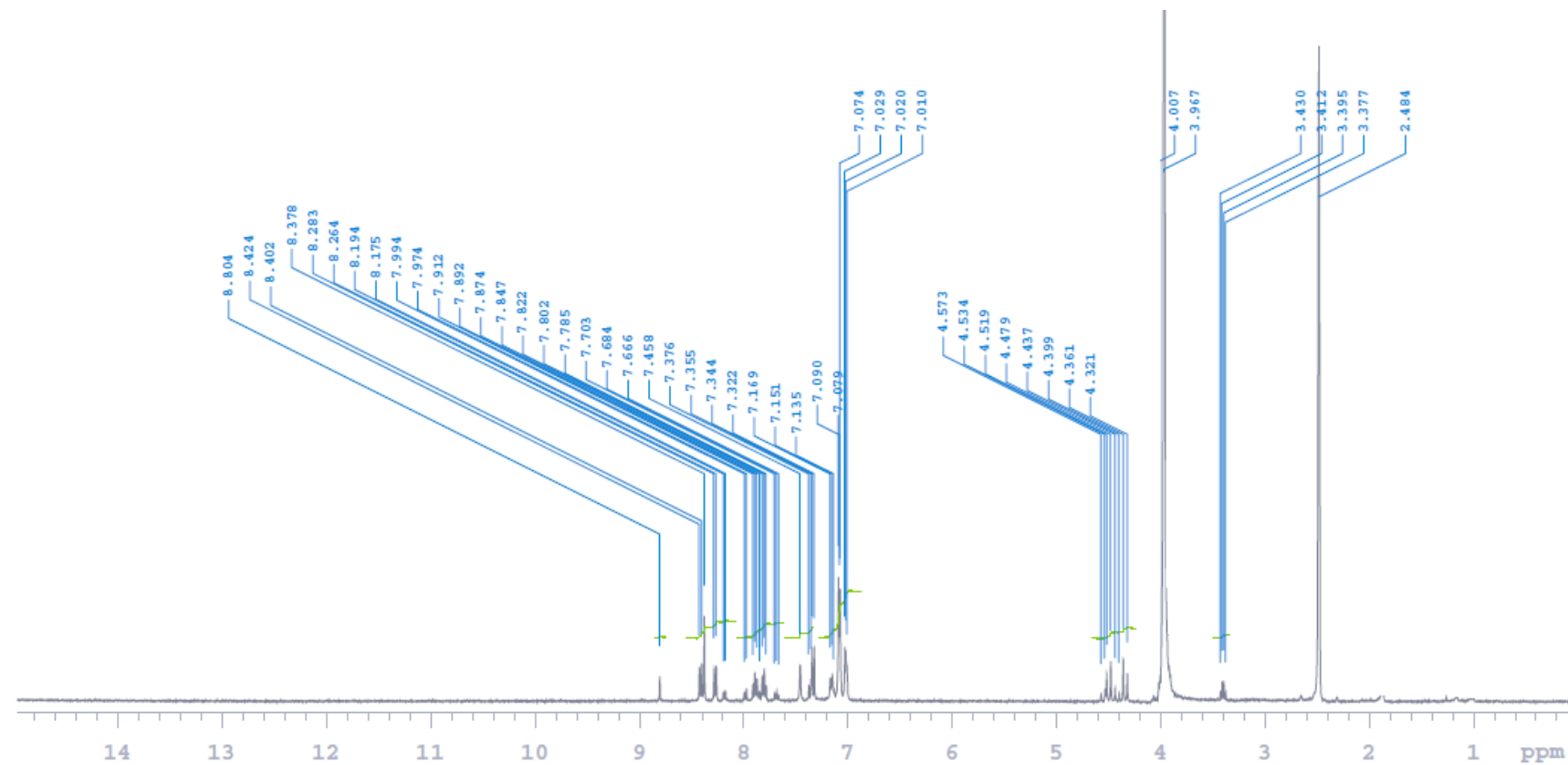

6c

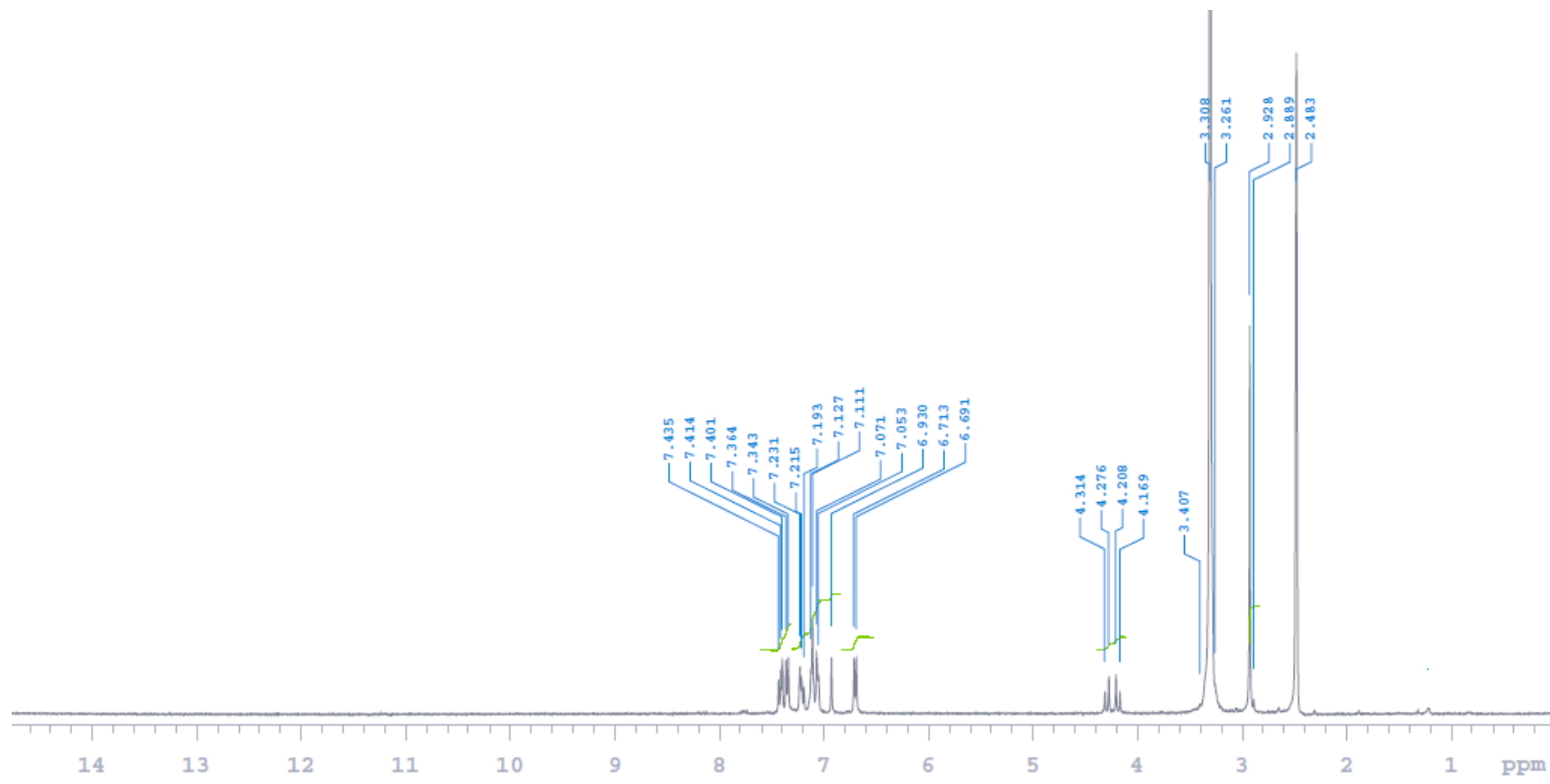

**6d**

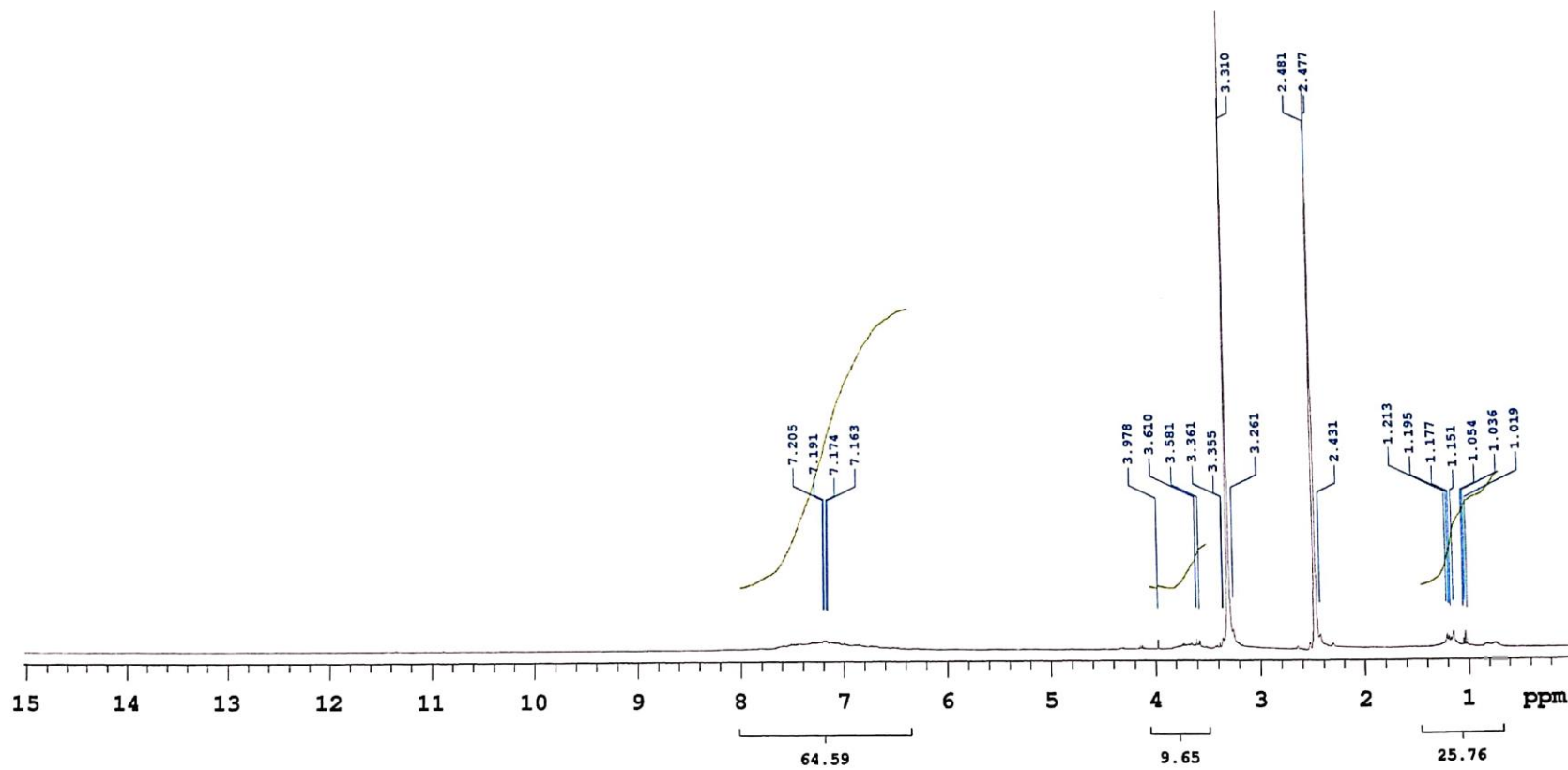

6e

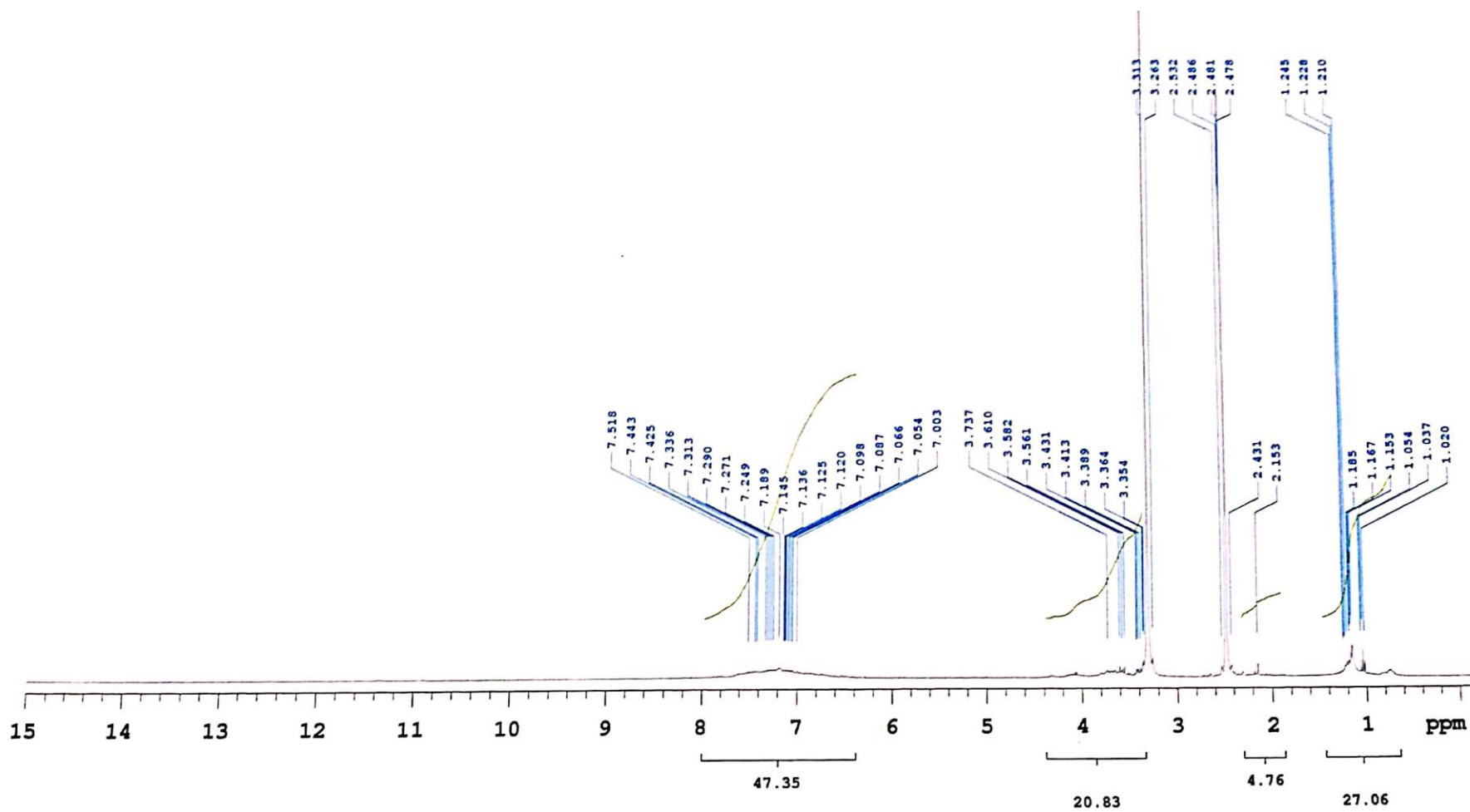

Supplement: Supplemental Material [file IENZ_A_1984904_SM4252.pdf]
